# Incidence and resistance rates of *Pseudomonas aeruginosa* bloodstream infections in Switzerland: a nationwide surveillance study (2010–2022)

**DOI:** 10.1007/s15010-024-02452-1

**Published:** 2025-01-30

**Authors:** Luzia Renggli, Andrea Burri, Simone Ehrhard, Michael Gasser, Andreas Kronenberg, The Swiss Centre for Antibiotic Resistance

**Affiliations:** 1 Swiss Centre for Antibiotic Resistance (ANRESIS), Institute for Infectious Diseases, University of Bern, Bern, Switzerland; 2 Department of Internal Medicine, Solothurner Spitäler, Spital Dornach, Dornach, Switzerland; 3 Department of Emergency Medicine, Inselspital, Bern University Hospital, University of Bern, Bern, Switzerland

**Keywords:** *Pseudomonas aeruginosa*, Bloodstream infections, Bacteremia, Incidence, Resistance rate, Switzerland

## Abstract

**Purpose:**

Bloodstream infections (BSIs) cause significant morbidity and mortality worldwide. *Pseudomonas aeruginosa* is an important microorganism in BSIs. The aim of this study was to analyze recent trends in the incidence and resistance rates of *P. aeruginosa* BSIs in Switzerland and its different linguistic regions.

**Methods:**

This retrospective, nationwide observational study analyzed the incidence (using Poisson regression models) and antimicrobial resistance (using logistic regression models) of *P. aeruginosa* BSIs in Switzerland from 2010 to 2022.

**Results:**

The annual incidence of *P. aeruginosa* BSIs in Switzerland increased from 5.5 BSIs per 100,000 inhabitants in 2010 to 7.6 BSIs per 100,000 inhabitants in 2022 (*p* < 0.001). The incidence was higher in the French-speaking region than in the German-speaking region. The resistance rates increased significantly for cefepime (2.4% in 2010, 8.8% in 2022; *p* < 0.001), ceftazidime (5.6% in 2010, 9.4% in 2022; *p* = 0.014), ciprofloxacin (3.3% in 2010, 6.5% in 2022; *p* = 0.014), and piperacillin-tazobactam (6.4% in 2010, 11.2% in 2022; *p* = 0.002). No significant trends were observed for carbapenem-, aminoglycoside-, or multidrug-resistant *P. aeruginosa*. A high incidence was observed in patients ≥ 80 years, whereas resistance rates were high in young patients.

**Conclusion:**

The increase in the incidence of *P. aeruginosa* BSIs emphasizes the importance of monitoring resistant and susceptible *P. aeruginosa* BSIs. Compared to the population-weighted mean resistance rates in Europe in 2022, those in Switzerland were lower, but an increase was observed for most antibiotics. The high resistance rates in young patients require further investigation.

**Supplementary Information:**

The online version contains supplementary material available at 10.1007/s15010-024-02452-1.

## Introduction

Bloodstream infections (BSIs) cause significant morbidity and mortality, and the incidence of these infections is increasing worldwide [1, 2]. The prognosis of BSIs depends on the type of pathogen, appropriate and early antimicrobial treatment, and the patient’s underlying disease [1, 3]. *Pseudomonas aeruginosa* is common in healthcare-associated infections and causes various device-associated infections [4, 5]. In intensive care units, *P. aeruginosa* has become one of the most common Gram-negative pathogens [6–8]. In Spain, for example, *P. aeruginosa* caused 5.5% of hospital-acquired BSIs in 2016 but was rare (1.4%) among community-acquired BSIs [9]. Among those with community-acquired *P. aeruginosa* BSIs, the main sources of infection were urinary tract infections (33.3%) and respiratory tract infections (16.7%), followed by intra-abdominal infections (11.6%) and infections related to vascular catheters (7.7%) [10].

In Europe, antimicrobial resistance surveillance has shown decreasing or stable trends in the resistance rates of invasive *P. aeruginosa* isolates between 2018 and 2022 for all antibiotics except piperacillin-tazobactam [11]. In contrast, in Switzerland, an increase in the resistance rates of invasive *P. aeruginosa* infections was reported in the Swiss Antibiotic Resistance Report [12]. In addition, an increase in the incidence of overall BSIs was observed in Switzerland from 2008 to 2014 [13].

Risk factors for *P. aeruginosa* BSIs, especially multidrug-resistant (MDR) strains, are severe immunodeficiency, malignancy, recent hospitalization, prior antibiotic treatment, and invasive devices [10, 14, 15]. Therapy for *P. aeruginosa* BSIs is challenging due to the high degree of resistance mechanisms, both acquired and intrinsic [14, 16, 17]. Early and appropriate antimicrobial treatment of invasive *P. aeruginosa* infections is crucial for ensuring patient outcomes [18]. Therefore, it is important for clinicians to know the regional incidence and resistance rates of *P. aeruginosa* infections. Population-based studies are considered the gold standard for defining the epidemiology of infectious diseases [19]. Nevertheless, worldwide, there are few detailed population-based data on the epidemiology of *P. aeruginosa* BSIs [20–24], and to our knowledge, to date, there are no population-based studies on the current incidence of *P. aeruginosa* BSIs in Switzerland. Therefore, this study aimed to analyze the overall incidence and resistance rates of *P. aeruginosa* BSIs in Switzerland and in different Swiss linguistic regions over a 13-year period.

## Methods

### Design and study population

This retrospective observational study was conducted in Switzerland over a period of 13 years (2010–2022). Cases of *P. aeruginosa* BSIs were included from laboratories of 78 Swiss acute care hospitals that reported data to the Swiss Center for Antimicrobial Resistance (ANRESIS) in 2010 and 2022 and for more than half of the years within this period. All five university hospitals in Switzerland were reporting data to ANRESIS and were included in the study.

### Data collection and processing

The *P. aeruginosa* BSI data were obtained from the ANRESIS database [25]. The participating laboratories are distributed all over Switzerland and accredited by national authorities. Deduplication was performed by counting only one isolate per patient and year– more precisely, in each case, the most resistant isolate.

First, the overall incidence of *P. aeruginosa* BSIs per 100,000 inhabitants in Switzerland was analyzed by extrapolating the number of cases per 100,000 inhabitants by the coverage of the included hospitals, assuming that all patients with *P. aeruginosa* BSIs are hospitalized. In 2010, the overall coverage in Switzerland was 59.0%, in 2022 59.3% and in 2017, the median year in the study period, the coverage was 58.1%. The calculation was based on the yearly bed-days of the 78 included acute care hospitals covered by ANRESIS. With the abovementioned inclusion criteria for hospitals, regularly delivering data within the study period, the variability of reporting hospitals was low, and therefore, the coverage in the median year was used throughout the whole study period.

Second, the incidence in the three main linguistic regions was calculated. In 2017, the coverage of yearly bed-days in the French-speaking region was 74.2%, the coverage in the German-speaking region was 52.1% and the coverage in the Italian-speaking region was 71.3%.

Third, the resistance rates and incidences of six antibiotics/antibiotic categories and MDR strains, including aminoglycosides, carbapenems, ceftazidime, cefepime, ciprofloxacin, and piperacillin-tazobactam were analyzed. Resistance was defined according to the ANRESIS-restricted definition [26]. In detail, for aminoglycosides, the entire category was considered resistant if *P. aeruginosa* was resistant to amikacin and/or gentamicin. The carbapenem category was considered resistant if *P. aeruginosa* was resistant to imipenem and/or meropenem. MDR was defined as resistance to three or more of five antibiotics/antibiotic categories [26]. For this definition, ceftazidime and cefepime were combined into one category.

Descriptive subgroup analyses were performed to assess the incidence and resistance rates in different age groups (< 2 years, 2–24 years, 25–49 years, 50–64 years, 65–79 years, and ≥ 80 years) and for males and females.

The yearly number of inhabitants in Switzerland and in each linguistic region was used as the denominator and was adjusted for sex and age group in the corresponding analyses [27]. In addition, a pooled resistance rate over the whole study period was calculated for each age group.

During the study period, the guidelines for antibiotic susceptibility testing and breakpoints changed from the Clinical & Laboratory Standards Institute (CLSI) to the European Committee on Microbial Susceptibility Testing (EUCAST), which is used by all but one laboratory. However, breakpoints for the definition of resistant *P. aeruginosa* did not change or changed only marginally and should not have affected the results [28].

In an additional post hoc analysis, the percentage of *P. aeruginosa* BSIs among all BSIs was calculated for each year of the study period. If *P. aeruginosa* was detected in at least one blood culture, the patient was considered to have a BSI with *P. aeruginosa.*

### Statistical analysis

First, temporal trends in the incidence of *P. aeruginosa* BSIs per 100,000 inhabitants were analyzed for Switzerland overall by fitting separate Poisson regression models for overall incidence and resistant isolates against aminoglycosides, carbapenems, cefepime, ceftazidime, ciprofloxacin, piperacillin-tazobactam and MDR *P. aeruginosa*. In a second step, possible trends in the different Swiss linguistic regions were considered by fitting Poisson regression models including the predictor variables year, linguistic region and the interaction between year and linguistic region.

Furthermore, logistic regression models were used to analyze the trends in the percentage of isolates resistant to aminoglycosides, carbapenems, cefepime, ceftazidime, ciprofloxacin, piperacillin-tazobactam and MDR *P. aeruginosa* among all *P. aeruginosa* BSIs in Switzerland. In addition, possible trends in resistance rates in the different Swiss linguistic regions were considered by applying multiple logistic regression models, including the predictor variables year, linguistic region and the interaction between year and linguistic region.

The results were considered significant if the *p* values of the likelihood ratio test and the *t* test of the explanatory variable were less than 0.05. Due to the small sample size in the Italian-speaking region, the results from the trend analyses including the Italian-speaking region are reported in the supplementary information only, to avoid misleading information. All analyses were performed using R software (version 4.2.2., R Core Team, Vienna, Austria).

## Results

### Overall incidence of *P. aeruginosa* BSIs

**Fig. 1:**
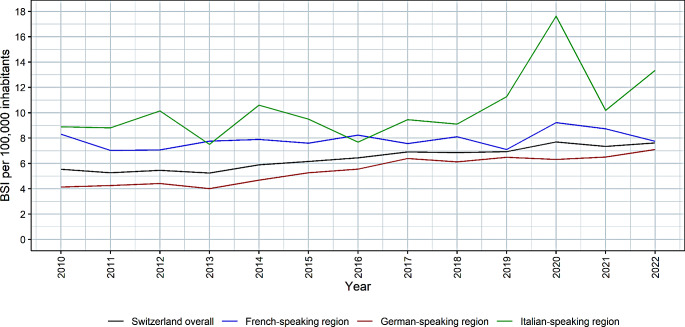
Incidence of *Pseudomonas aeruginosa* bloodstream infections in Switzerland and its different linguistic regions, 2010–2022

During the study period, a total of 4,934 *P. aeruginosa* BSIs were analyzed. In 2010, the incidence of *P. aeruginosa* BSIs was 5.5 per 100,000 inhabitants and increased significantly (*p* < 0.001) over the 13 years to 7.6 per 100,000 inhabitants in 2022 (Fig. 1; Table 1).

**Table 1:** Temporal course of the incidence and percentage of resistant *Pseudomonas aeruginosa* bloodstream infections, Switzerland, 2010–2022

*P*. aeruginosa BSIs	2010	2022	Trend
**Incidence overall** [per 100,000 inhabitants]	5.539	7.621	↑ (*p* < 0.001)
**Incidence of resistant isolates** [per 100,000 inhabitants]			
Aminoglycosides	0.072	0.13	-
Carbapenems	0.505	0.682	↑ (*p* < 0.001)
Cefepime	0.108	0.65	↑ (*p* < 0.001)
Ceftazidime	0.307	0.699	↑ (*p* < 0.001)
Ciprofloxacin	0.18	0.487	↑ (*p* < 0.001)
Piperacillin-tazobactam	0.343	0.812	↑ (*p* < 0.001)
MDR	0.126	0.26	↑ (*p* = 0.001)
**Percentage of resistant isolates** [%]			
Aminoglycosides	1.5	2	-
Carbapenems	9.2	9.1	-
Cefepime	2.4	8.8	↑ (*p* < 0.001)
Ceftazidime	5.6	9.4	↑ (*p* = 0.014)
Ciprofloxacin	3.3	6.5	↑ (*p* = 0.014)
Piperacillin-tazobactam	6.4	11.2	↑ (*p* = 0.002)
MDR	2.3	3.5	-

BSIs; bloodstream infections, MDR; multidrug-resistant, ↑; increasing trend

A comparison of the different linguistic regions in Switzerland revealed that the incidence was significantly (*p* < 0.001) lower in the German-speaking region than in the French-speaking region. However, the increase in incidence was significantly (*p* < 0.001) greater in the German-speaking region than in the French-speaking region, where the incidence remained stable at approximately 8 BSIs per 100,000 inhabitants (Fig. 1, Supplementary Table 9). In the German-speaking region, the incidence of *P. aeruginosa* BSIs increased from 4.1 per 100,000 inhabitants in 2010 to 7.1 per 100,000 inhabitants in 2022. The incidence in the Italian-speaking region was 8.9 per 100,000 inhabitants in 2010 and increased to 13.4 per 100,000 inhabitants in 2022, but the sample sizes were small in this region, and a large variance was observed (Fig. 1, Supplementary Table 3).

The incidence was more than twofold higher in male patients than in female patients every year except in 2017, and an increase in incidence was observed in both sexes (Supplementary Fig. 1 and Table 7).

### Incidence of resistant isolates

For all antibiotics (carbapenems, cefepime, ciprofloxacin, ceftazidime, piperacillin-tazobactam and MDR *P. aeruginosa*) except for aminoglycosides, the incidence of resistant isolates increased significantly according to the corresponding Poisson regression models (Table 1, Supplementary Fig. 3A and 3B). Statistically significant differences between the linguistic regions were observed for the incidence of carbapenem-resistant *P. aeruginosa* BSIs only; the incidence was higher in the French-speaking region than in the German-speaking region (*p* = 0.036), while the increase was greater in the German-speaking region than in the French-speaking region (*p* = 0.037, Supplementary Table 9).

### Resistance rates

During the study period, increasing resistance rates were observed for cefepime (2.4% in 2010 to 8.8% in 2022, *p* < 0.001), ceftazidime (5.6% in 2010 to 9.4% in 2022, *p* = 0.014), ciprofloxacin (3.3% in 2010 to 6.5% in 2022, *p* = 0.014), and piperacillin-tazobactam (6.4% in 2010 to 11.2% in 2022, *p* = 0.002). There was no significant change in the resistance rates for carbapenems (9.2% in 2010, 9.1% in 2022), aminoglycosides (1.5% in 2010, 2.0% in 2022) or MDR *P. aeruginosa* (2.3% in 2010 and 3.5% in 2022, Fig. 2; Table 1).

**Fig. 2:**
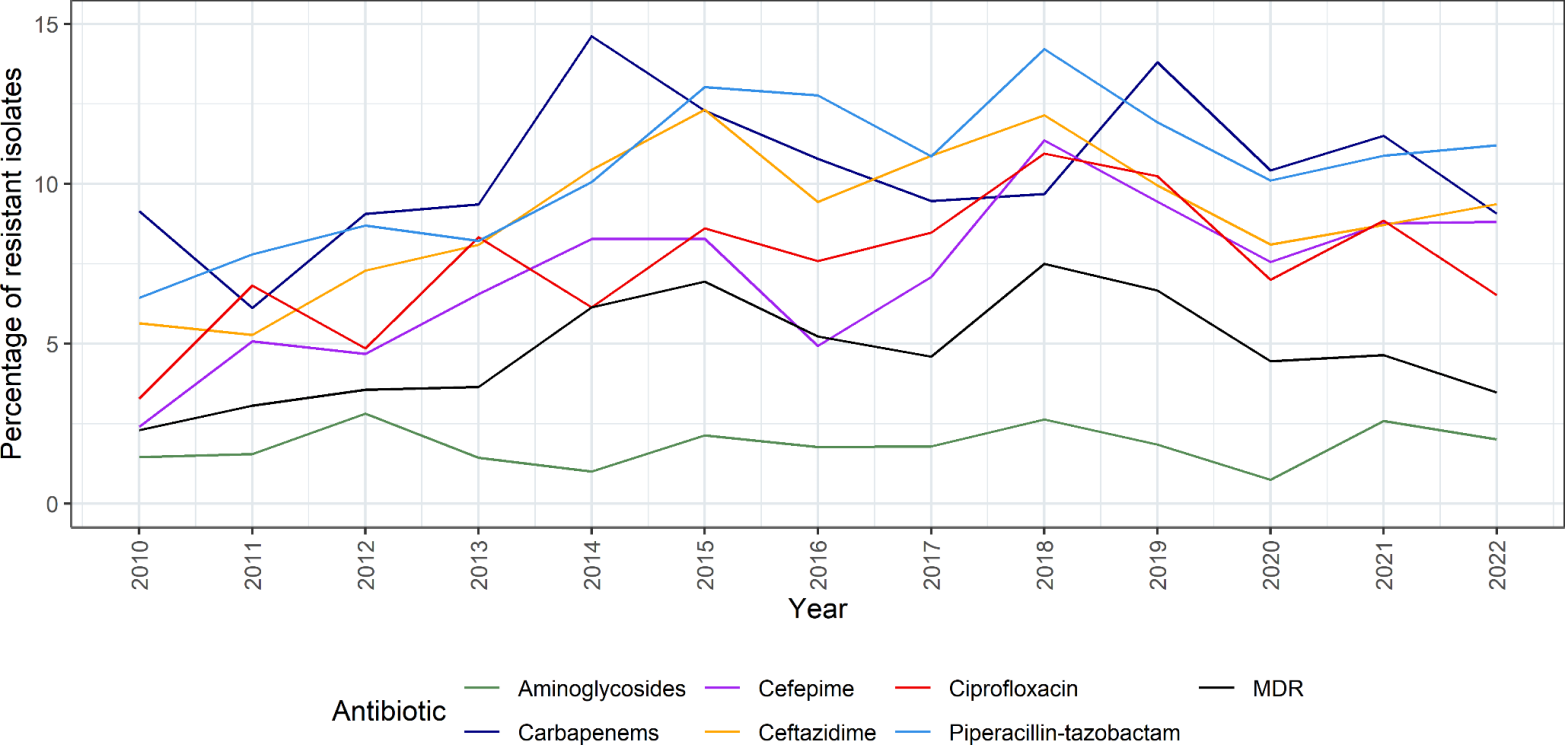
Percentage of resistant *Pseudomonas aeruginosa* bloodstream infections in Switzerland, 2010–2022

The resistance rates for carbapenems were higher in the French-speaking region than in the German-speaking region of Switzerland every year except in 2018 (the yearly resistance rates were 0.9−6.7% higher in the French-speaking region), whereas for the other tested antibiotics, no differences between the linguistic regions were observed (Supplementary Fig. 3A and Table 4).

### Age group analysis

**Fig. 3:**
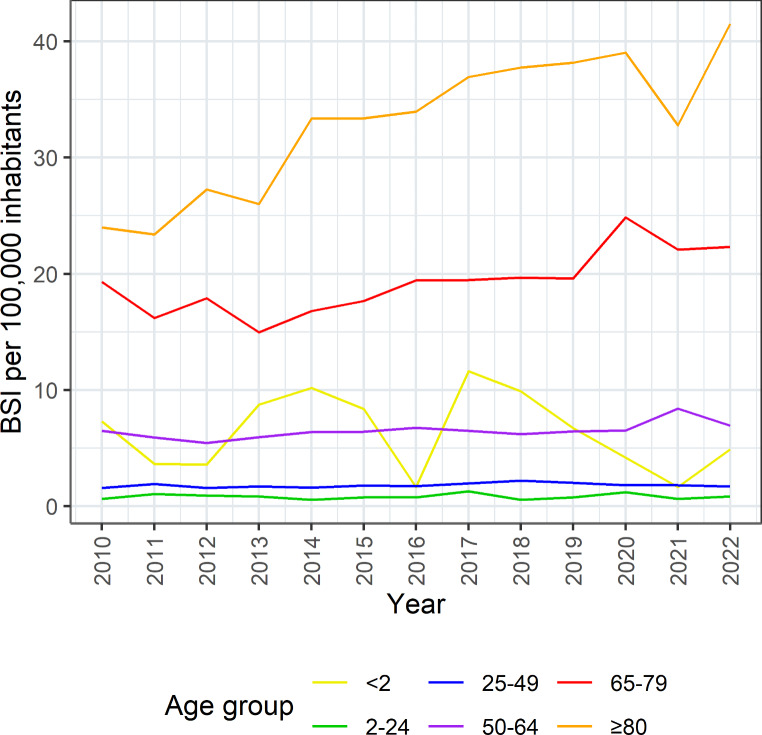
Incidence of *Pseudomonas aeruginosa* bloodstream infections per age group, Switzerland, 2010–2022

Among the different age groups, the incidence was highest in patients ≥ 80 years: 24.0 per 100,000 inhabitants in 2010, with an increase up to 41.5 per 100,000 inhabitants in 2022 (Fig. 3, Supplementary Table 5). A less pronounced increase was observed in the 65–79 years age group, with an incidence of 19.3 per 100,000 inhabitants in 2010 and an increase up to 22.3 per 100,000 inhabitants in 2022. The lowest incidence was observed in patients 2–24 years old (0.6 per 100,000 inhabitants in 2010 and 0.8 per 100,000 inhabitants in 2022). Infants and toddlers up to 2 years of age had an incidence of 7.3 per 100,000 inhabitants in 2010 and 4.9 per 100,000 inhabitants in 2022, with small absolute numbers (4 cases in 2010 and 6 cases in 2022) and a high variance. No major changes were observed in the other age groups.

**Fig. 4:**
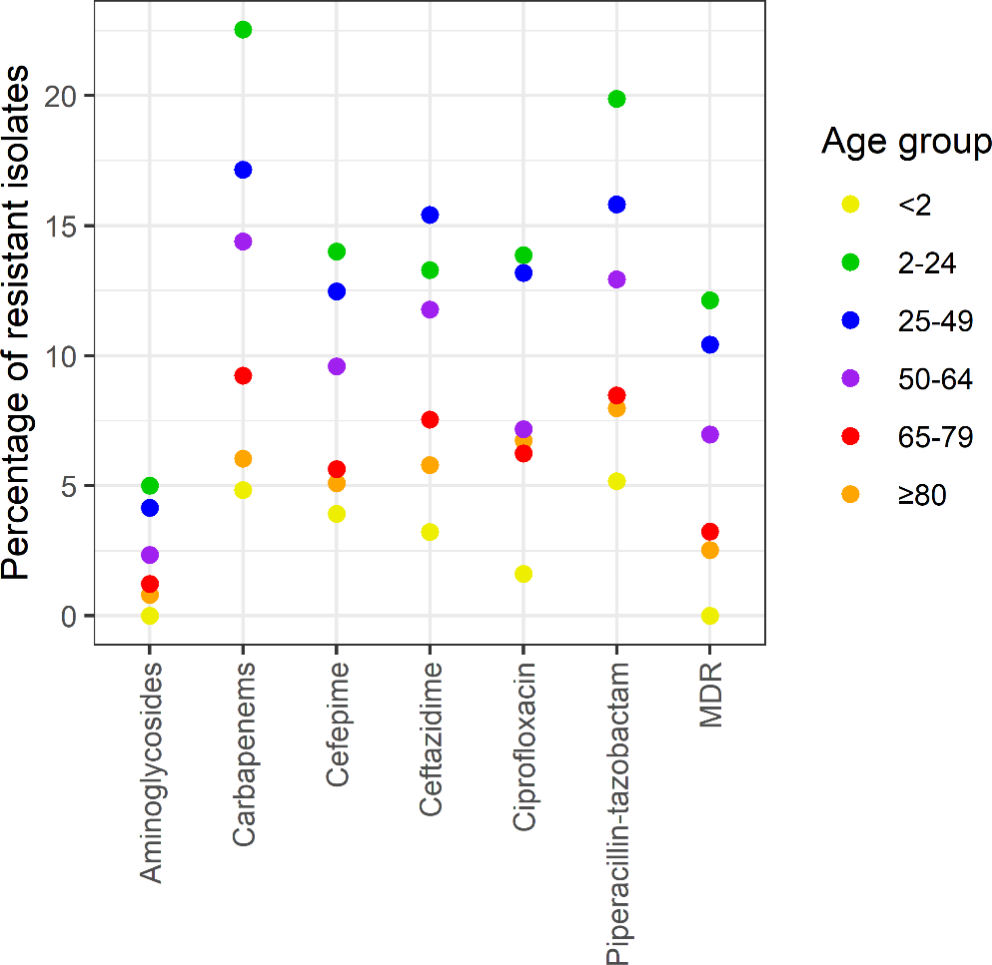
Percentage of resistant *Pseudomonas aeruginosa* bloodstream infections per age group, Switzerland; pooled 2010–2022

The pooled resistance rates varied among the different age groups. High resistance rates were observed in patients aged 2–24 years, low resistance rates were observed in patients < 2 years old and patients ≥ 65 years old (Fig. 4).

### Additional post hoc analysis

Among all BSIs, 3% of the BSIs were caused by *P. aeruginosa*. This percentage remained stable during the study period (Supplementary Fig. 4). The percentage of *P. aeruginosa* among all BSIs was slightly higher in the French-speaking region than in the German-speaking region in every year except in 2017, when the percentages were equal. In children up to 2 years, the percentage of *P. aeruginosa* among all BSIs was less than 1.5%; in patients aged 2–24 years, the annual range was 1.5–4%; and in patients over 25 years, the percentage was between 2.5% and 4% (Supplementary Fig. 5).

## Discussion

The main finding of this study was an increase in the incidence of *P. aeruginosa* BSIs in Switzerland from 2010 to 2022. This finding is in line with previous studies and suggests a possible continuation of the previously reported trends describing an increase in BSIs caused by all pathogens in Switzerland (2008–2014) [13]. Indeed, the percentage of *P. aeruginosa* BSIs compared to all BSIs was 3% and remained stable during the study period. Since the most recent Swiss point prevalence survey did not report a significant change in healthcare-associated infections (HAIs) between 2017 and 2022 and since catheter-associated urinary tract infections (CAUTIs) decreased in Switzerland, changes in HAIs or CAUTI rates do not seem to explain the increasing incidence of *P. aeruginosa* BSIs [29–31]. The incidence was particularly high in older people, which was also found in other studies in Europe and North America [20–23]. In this Swiss study, an increase in incidence was mainly observed in patients older than 80 years, whereas there were no or only small changes in other age groups. As the annual age-adjusted incidence was calculated, changes in demographics should not have had any impact on these results. A recent study in Canada also revealed a high incidence of BSIs in older people, predominantly consisting of Gram-negative bacteremia, and urinary tract infections as a common source [32]. That study revealed no increase in incidence from 2010 to 2020 after age adjustment, whereas other studies have shown increasing trends in the incidence of BSIs in older patients [13, 24, 33]. The rising life expectancy, which is associated with an increase in frailty in older people, might partly explain these findings. Furthermore, with the increasing use of immunosuppressive therapies for cancer and autoimmune diseases, it is suspected that an increasing percentage of the population is immunocompromised. In Switzerland, the number of inhabitants treated with cancer or immune system drugs increased from 149,364 inhabitants in 2010 (1.90% of the total population) to 323,901 inhabitants in 2022 (3.67% of the total population) [27, 34]. Considering that severe immunodeficiency is a risk factor for *P. aeruginosa* BSI [10, 14, 15], this might contribute to the increasing incidence of *P. aeruginosa* BSIs.

Compared to the European Union (EU)/European Economic Area (EEA) population-weighted mean resistance rates of *P. aeruginosa* in 2022 [11], the resistance rates in Switzerland were relatively low. However, in recent years, the mean resistance rates in EU/EAA countries have remained stable or trended downward (except for a recent increase in the piperacillin-tazobactam resistance rate) [11], whereas the trend analysis from this study revealed an upward trend for most antibiotics in Switzerland from 2010 to 2022. On closer inspection, in this study, a decrease in resistance rates can be observed since 2018, and further monitoring to verify this trend is needed.

This study revealed differing trends in incidence and resistance rates in the linguistic regions of Switzerland. The initially lower incidence of overall *P. aeruginosa* BSIs in the German-speaking region was approaching the higher incidence in the French- and Italian-speaking regions. The same trend was observed in a recent study conducted on *Staphylococcus aureus* BSIs in Switzerland [33]. The analysis of carbapenem resistance revealed an increasing incidence of carbapenem-resistant *P. aeruginosa* BSIs, but a stable percentage of carbapenem-resistance from 2010 until 2022. Therefore, the increasing incidence of carbapenem-resistant *P. aeruginosa* BSIs can be explained by an overall increase in incidence. The same trend was observed in the German-speaking region, which actually contributes most to the overall incidence, but not in the French-speaking region of Switzerland: Carbapenem resistance rates were higher in the French-speaking region of Switzerland than in the German-speaking region, but the incidence of carbapenem-resistant *P. aeruginosa* BSIs remained stable in the French-speaking region, whereas it increased in the German-speaking region, consistent with the trends for overall incidence. In France, carbapenem resistance rates were 17.8% in 2010 and decreased to 11.3% in 2022 [35–37]. Accordingly, carbapenem resistance rates in 2022 were comparable to those in the French-speaking region of Switzerland (11.4%). Growing migratory movements, especially from France to Switzerland, and a higher percentage of people with a migration background in the French- and Italian-speaking regions than in the German-speaking region, might explain the regional differences and the approximation of resistance rates to neighboring countries [38].

Another interesting finding of this study were the high resistance rates in young patients (2–24 years), whereas the resistance rates in older patients were relatively low. Possible reasons for these diverging results might be the comorbidities of patients in this age group (for example, cystic fibrosis); however, further investigation is needed to explain the high resistance rates in these young patients.

The main limitation of this study was the lack of information on the source of infection and comorbidities. In addition, data on whether the BSI was a nosocomial or community-acquired infection were not available. Therefore, it remains unclear whether the increase in incidence is driven by changes in hospital-acquired or community-acquired infections and possible outbreaks in specific hospitals to explain the high resistance rates in young people couldn’t be identified.

Another limitation is that not every laboratory in Switzerland reported BSIs to ANRESIS, and despite the inclusion criteria for hospitals regularly delivering data within the study period to favor consistent reporting, a possible variability of reporting hospitals might represent a bias. To calculate the nationwide incidence, the data had to be extrapolated, as previously described. The coverage, however, was relatively high and included all university hospitals, which is a main strength of this study. Another strength of this study is the specific investigation of the different linguistic regions of this heterogeneous country, as national, aggregated data might mask diverging regional trends.

In conclusion, the incidence of both resistant and susceptible *P. aeruginosa* BSIs increased in Switzerland from 2010 to 2022, especially in older patients and in the German-speaking region. The percentage of carbapenem-resistant *P. aeruginosa* BSIs was stable in Switzerland, but the incidence of carbapenem-resistant *P. aeruginosa* BSIs increased due to an increase in overall incidence. This emphasizes the importance of extending the monitoring of resistant to susceptible pathogens causing BSIs. The high resistance rates of *P. aeruginosa* in young patients with BSIs require additional detailed research, especially on comorbidities (for example, cystic fibrosis) in this age group.

## Electronic supplementary material

Below is the link to the electronic supplementary material.

Supplementary Material 1

## Data Availability

Data is provided within the manuscript or supplementary information file. All additional data can be made available upon request to the corresponding author.
