# Supplementary material for: Incidence and resistance rates of Pseudomonas aeruginosa bloodstream infections in Switzerland: a nationwide surveillance study (2010–2022)
Source: Infection. 2025 Jan 30;53(4):1373–81. doi: 10.1007/s15010-024-02452-1 (PMC12316723; doi:10.1007/s15010-024-02452-1)
Supplement: Supplementary file 1 — Supplementary Material 1 [file 15010_2024_2452_MOESM1_ESM.docx]

# Incidence and resistance rates of *Pseudomonas aeruginosa*

# bloodstream infections in Switzerland: a nationwide surveillance

# study (2010–2022)

Luzia Renggli^1*^, Andrea Burri^2*^, Simone Ehrhard^3^, Michael Gasser^1^, Andreas Kronenberg^1^, and the Swiss Centre for Antibiotic Resistance

*contributed equally

^1^Swiss Centre for Antibiotic Resistance (ANRESIS), Institute for Infectious Diseases, University of Bern, Bern, Switzerland

^2^Department of Internal Medicine, Solothurner Spitäler, Spital Dornach, Dornach, Switzerland

^3^Department of Emergency Medicine, Inselspital, Bern University Hospital, University of Bern, Bern, Switzerland

Corresponding author: Andreas Kronenberg, [andreas.kronenberg@unibe.ch](mailto:andreas.kronenberg@unibe.ch)

Journal: Infection

# Supplementary Figures


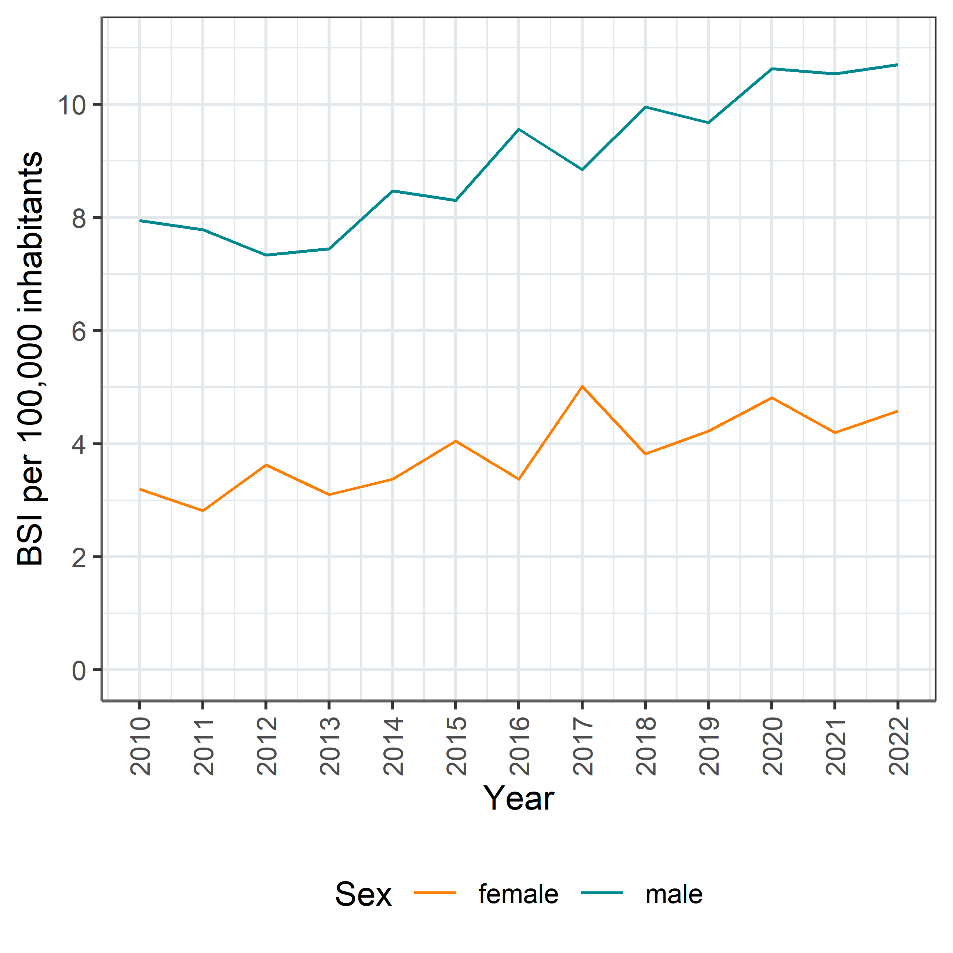


**Supplementary Fig. 1**: Incidence of *Pseudomonas aeruginosa* bloodstream infections per sex, Switzerland, 2010–2022


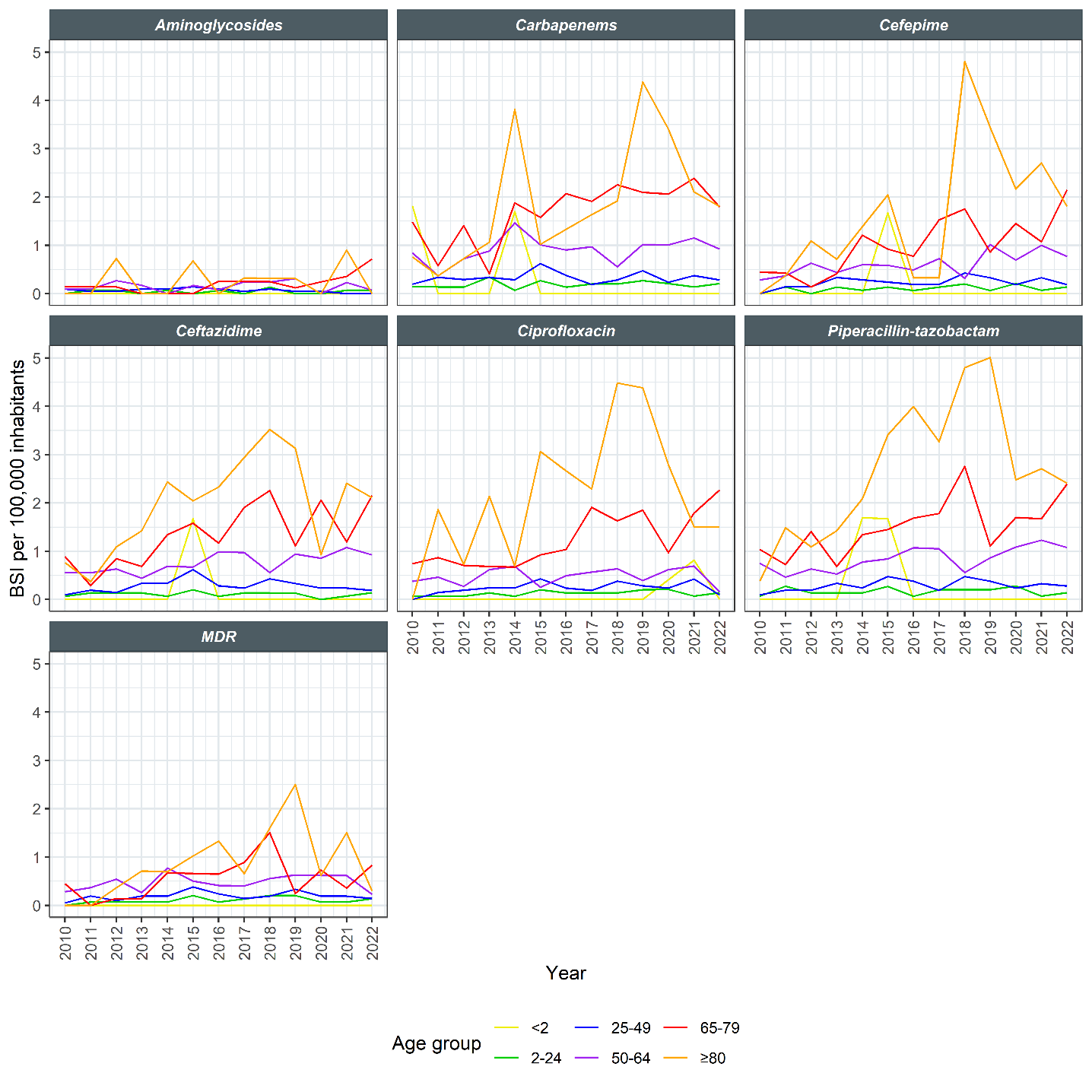


**Supplementary Fig. 2** Incidence of resistant *Pseudomonas aeruginosa* bloodstream infections per age group and antibiotic, Switzerland, 2010–2022


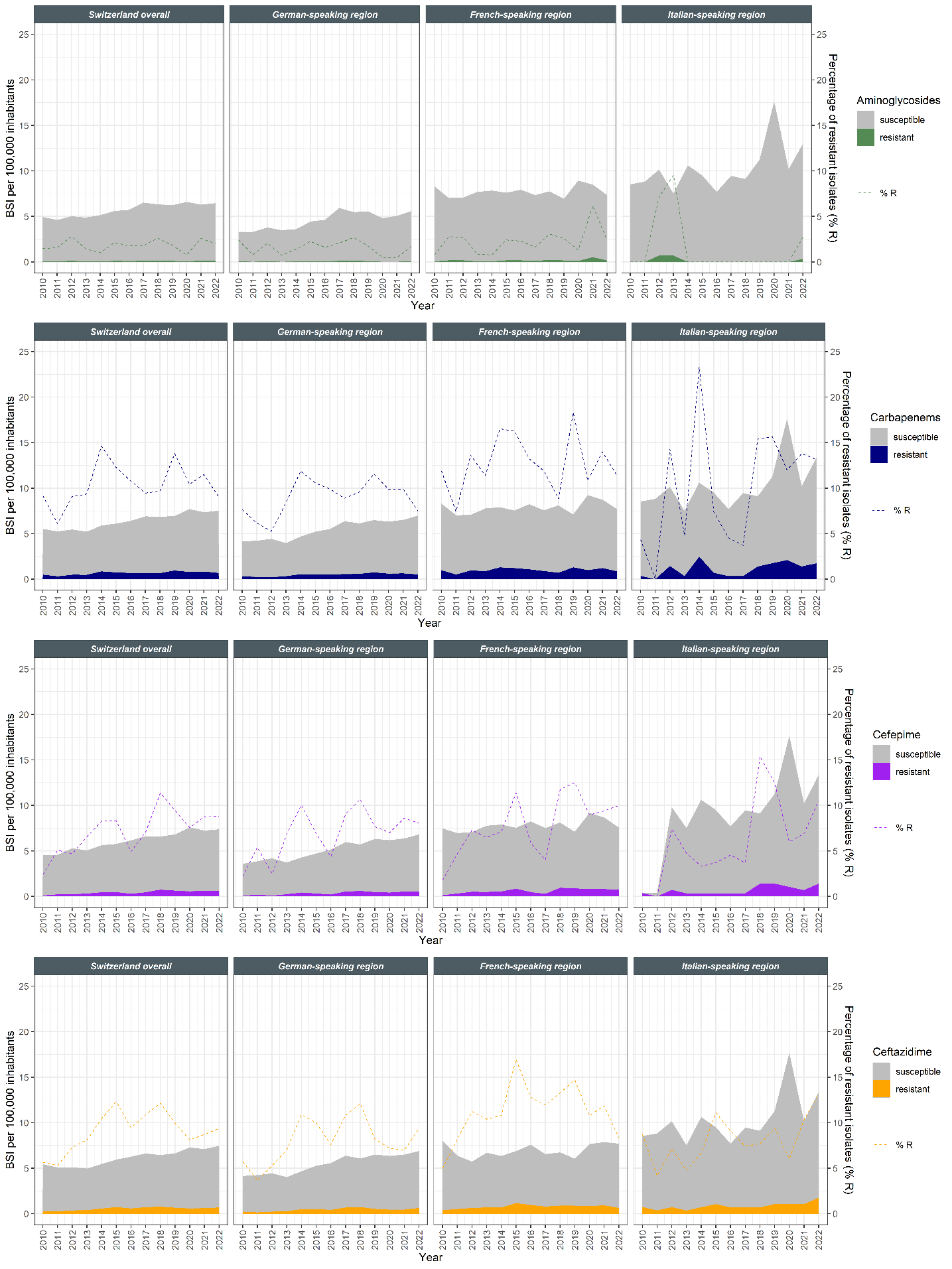


**Supplementary Fig. 3A** Incidence of susceptible and resistant *Pseudomonas aeruginosa* bloodstream infections and percentage of resistant isolates in the different Swiss linguistic regions 2010–2022


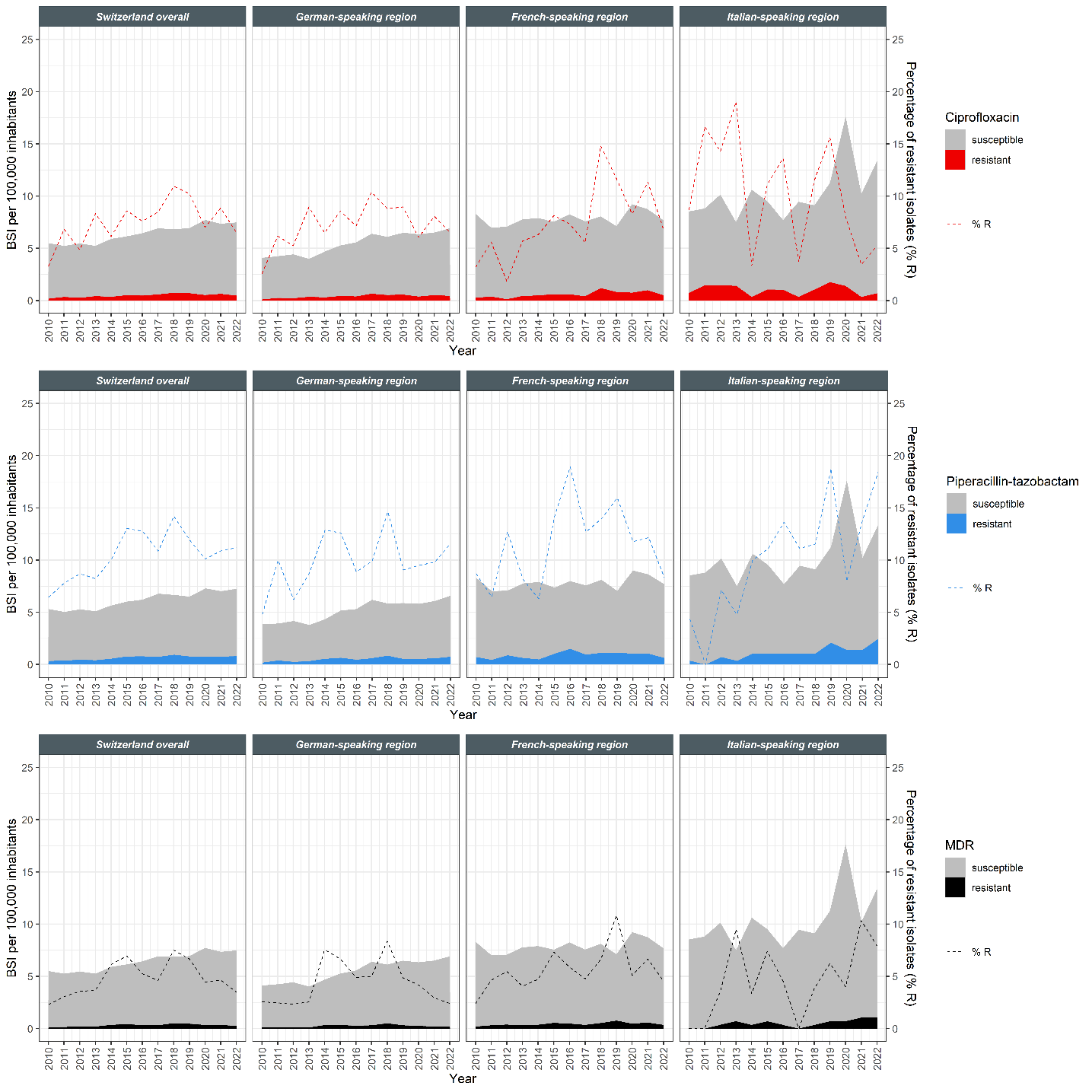


**Supplementary Fig. 3B** Incidence of susceptible and resistant *Pseudomonas aeruginosa* bloodstream infections and percentage of resistant isolates in the different Swiss linguistic regions 2010–2022


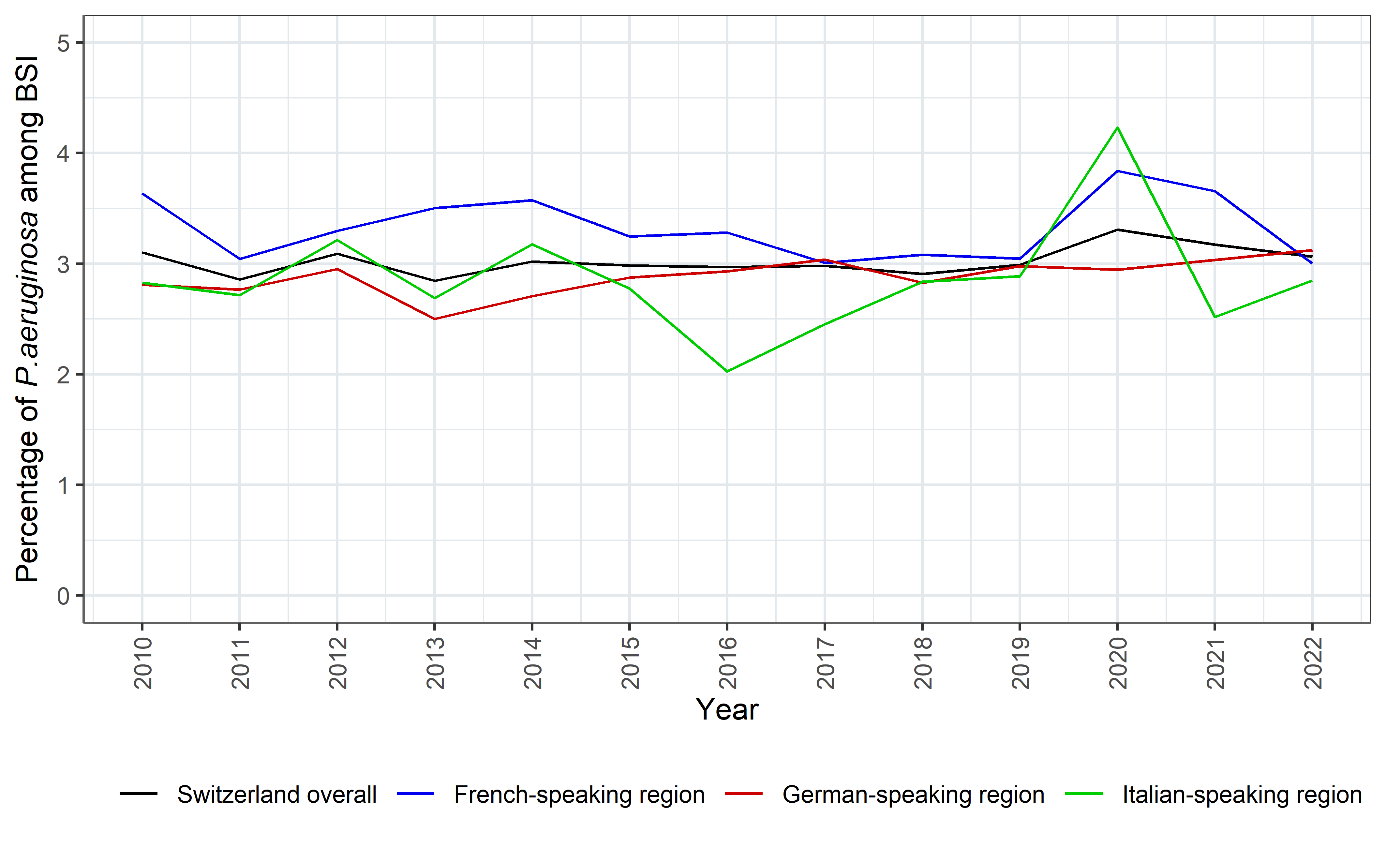


**Supplementary Fig. 4** Percentage of *Pseudomonas aeruginosa* bloodstream infections among all bloodstream infections in Switzerland and its different linguistic regions, 2010–2022


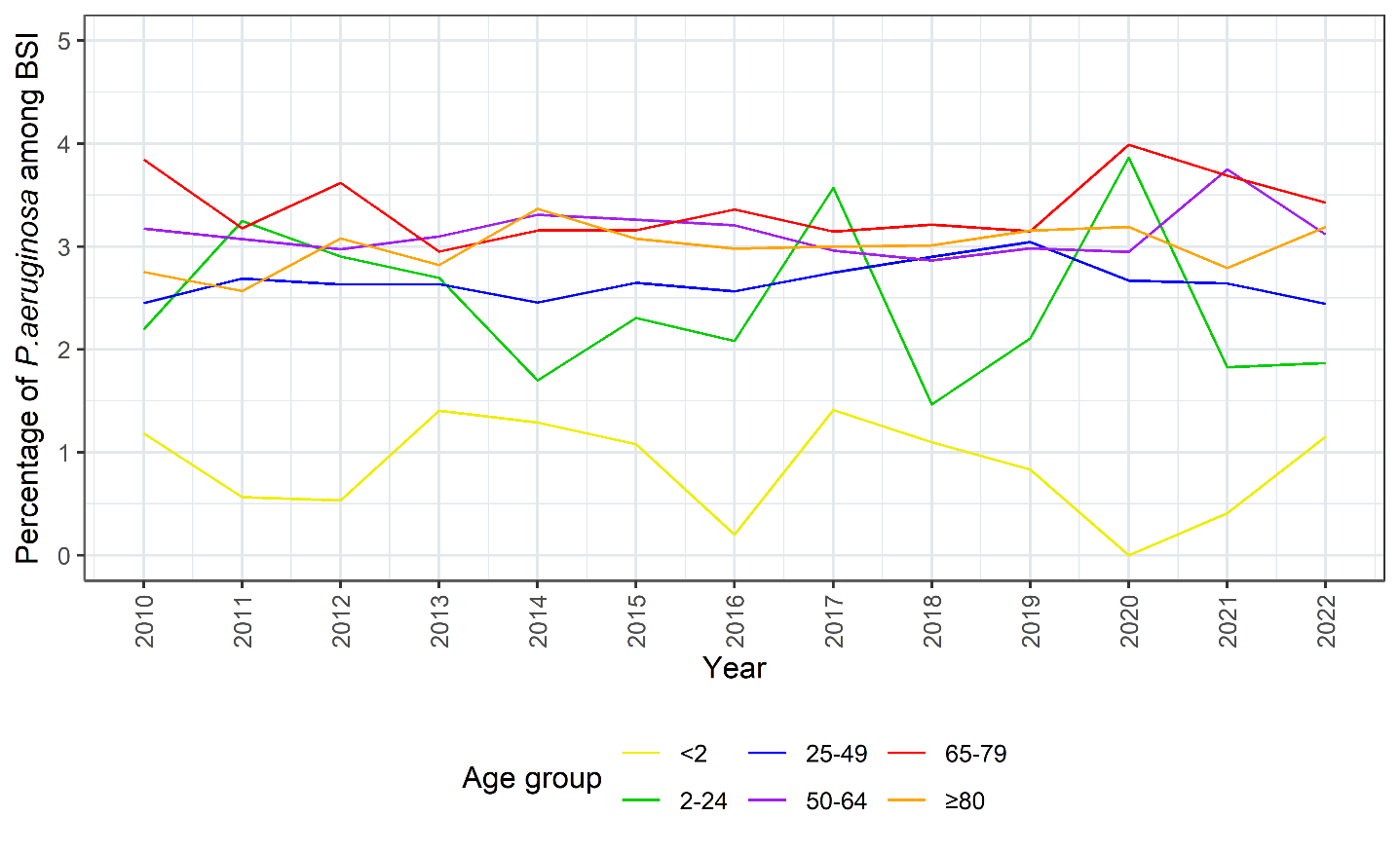


**Supplementary Fig. 5** Percentage of *Pseudomonas aeruginosa* bloodstream infections among all bloodstream infections per age group, 2010–2022

# Supplementary Tables

**Supplementary Table 1** Incidence of *Pseudomonas aeruginosa* bloodstream infections in Switzerland

|  | **2010** | **2011** | **2012** | **2013** | **2014** | **2015** | **2016** | **2017** | **2018** | **2019** | **2020** | **2021** | **2022** |
| --- | --- | --- | --- | --- | --- | --- | --- | --- | --- | --- | --- | --- | --- |
| Incidence [N per 100,000 inhabitants] | 5.539 | 5.266 | 5.458 | 5.251 | 5.895 | 6.156 | 6.443 | 6.912 | 6.864 | 6.93 | 7.698 | 7.345 | 7.621 |
| Number of samples tested | 307 | 295 | 309 | 301 | 342 | 361 | 382 | 413 | 413 | 420 | 470 | 452 | 469 |

**Supplementary Table 2** Resistant *Pseudomonas aeruginosa* bloodstream infections: percentage of resistant isolates (% R), incidence [N per 100,000 inhabitants], number of samples tested [N tested], Switzerland

| **Antibiotic** | **Unit** | **2010** | **2011** | **2012** | **2013** | **2014** | **2015** | **2016** | **2017** | **2018** | **2019** | **2020** | **2021** | **2022** |
| --- | --- | --- | --- | --- | --- | --- | --- | --- | --- | --- | --- | --- | --- | --- |
| Aminoglycosides | % R | 1.5 | 1.6 | 2.8 | 1.4 | 1 | 2.1 | 1.8 | 1.8 | 2.6 | 1.9 | 0.7 | 2.6 | 2 |
| Aminoglycosides | Incidence | 0.072 | 0.071 | 0.141 | 0.07 | 0.052 | 0.119 | 0.101 | 0.117 | 0.166 | 0.116 | 0.049 | 0.162 | 0.13 |
| Aminoglycosides | N tested | 274 | 258 | 284 | 279 | 298 | 328 | 339 | 390 | 380 | 378 | 402 | 387 | 397 |
| Carbapenems | % R | 9.2 | 6.1 | 9.1 | 9.4 | 14.6 | 12.3 | 10.8 | 9.5 | 9.7 | 13.8 | 10.4 | 11.5 | 9.1 |
| Carbapenems | Incidence | 0.505 | 0.321 | 0.495 | 0.488 | 0.862 | 0.75 | 0.691 | 0.653 | 0.665 | 0.957 | 0.803 | 0.845 | 0.682 |
| Carbapenems | N tested | 306 | 294 | 309 | 299 | 342 | 358 | 380 | 412 | 413 | 420 | 470 | 452 | 463 |
| Cefepime | % R | 2.4 | 5.1 | 4.7 | 6.6 | 8.3 | 8.3 | 4.9 | 7.1 | 11.4 | 9.4 | 7.6 | 8.8 | 8.8 |
| Cefepime | Incidence | 0.108 | 0.232 | 0.247 | 0.331 | 0.465 | 0.477 | 0.304 | 0.469 | 0.748 | 0.644 | 0.573 | 0.634 | 0.65 |
| Cefepime | N tested | 250 | 256 | 299 | 290 | 326 | 338 | 365 | 395 | 396 | 413 | 463 | 445 | 454 |
| Ceftazidime | % R | 5.6 | 5.3 | 7.3 | 8.1 | 10.4 | 12.3 | 9.4 | 10.9 | 12.1 | 10 | 8.1 | 8.7 | 9.4 |
| Ceftazidime | Incidence | 0.307 | 0.268 | 0.371 | 0.401 | 0.569 | 0.733 | 0.59 | 0.72 | 0.781 | 0.66 | 0.59 | 0.617 | 0.699 |
| Ceftazidime | N tested | 301 | 284 | 288 | 284 | 316 | 349 | 371 | 395 | 387 | 402 | 444 | 436 | 459 |
| Ciprofloxacin | % R | 3.3 | 6.8 | 4.9 | 8.3 | 6.1 | 8.6 | 7.6 | 8.5 | 10.9 | 10.2 | 7 | 8.8 | 6.5 |
| Ciprofloxacin | Incidence | 0.18 | 0.357 | 0.265 | 0.436 | 0.362 | 0.529 | 0.489 | 0.586 | 0.748 | 0.71 | 0.54 | 0.65 | 0.487 |
| Ciprofloxacin | N tested | 304 | 293 | 309 | 300 | 342 | 360 | 382 | 413 | 411 | 420 | 471 | 452 | 460 |
| Piperacillin-tazobactam | % R | 6.4 | 7.8 | 8.7 | 8.2 | 10.1 | 13 | 12.8 | 10.9 | 14.2 | 11.9 | 10.1 | 10.9 | 11.2 |
| Piperacillin-tazobactam | Incidence | 0.343 | 0.393 | 0.459 | 0.419 | 0.569 | 0.784 | 0.793 | 0.736 | 0.947 | 0.776 | 0.737 | 0.764 | 0.812 |
| Piperacillin-tazobactam | N tested | 295 | 282 | 299 | 292 | 328 | 353 | 368 | 405 | 401 | 394 | 445 | 432 | 446 |
| MDR | % R | 2.3 | 3.1 | 3.6 | 3.7 | 6.1 | 6.9 | 5.2 | 4.6 | 7.5 | 6.7 | 4.5 | 4.6 | 3.5 |
| MDR | Incidence | 0.126 | 0.161 | 0.194 | 0.192 | 0.362 | 0.426 | 0.337 | 0.318 | 0.515 | 0.462 | 0.344 | 0.341 | 0.26 |
| MDR | N tested | 305 | 294 | 309 | 301 | 342 | 360 | 382 | 413 | 413 | 420 | 471 | 452 | 460 |

MDR; multidrug-resistant

**Supplementary Table 3** Incidence of *Pseudomonas aeruginosa* bloodstream infections stratified by Swiss linguistic regions

| **Linguistic region** | **2010** | **2011** | **2012** | **2013** | **2014** | **2015** | **2016** | **2017** | **2018** | **2019** | **2020** | **2021** | **2022** |
| --- | --- | --- | --- | --- | --- | --- | --- | --- | --- | --- | --- | --- | --- |
| **Incidence**  [N per 100,000 inhabitants] |  |  |  |  |  |  |  |  |  |  |  |  |  |
| French-speaking region | 8.31 | 7.025 | 7.069 | 7.765 | 7.894 | 7.603 | 8.24 | 7.568 | 8.111 | 7.107 | 9.226 | 8.737 | 7.746 |
| German-speaking region | 4.14 | 4.257 | 4.423 | 4.018 | 4.684 | 5.263 | 5.552 | 6.394 | 6.126 | 6.489 | 6.319 | 6.508 | 7.104 |
| Italian-speaking region | 8.9 | 8.815 | 10.144 | 7.503 | 10.606 | 9.503 | 7.69 | 9.454 | 9.112 | 11.269 | 17.629 | 10.187 | 13.349 |
| **Number of samples** |  |  |  |  |  |  |  |  |  |  |  |  |  |
| French-speaking region | 126 | 108 | 110 | 123 | 127 | 124 | 136 | 126 | 136 | 120 | 157 | 150 | 133 |
| German-speaking region | 157 | 163 | 171 | 157 | 185 | 210 | 224 | 260 | 251 | 268 | 263 | 273 | 298 |
| Italian-speaking region | 24 | 24 | 28 | 21 | 30 | 27 | 22 | 27 | 26 | 32 | 50 | 29 | 38 |

**Supplementary Table 4** Resistant *Pseudomonas aeruginosa* bloodstream infections, incidence [N per 100,000 inhabitants] and percentage of resistant isolates [% R] stratified by Swiss linguistic regions

| **Antibiotic** | **Unit** | **Linguistic region** | **2010** | **2011** | **2012** | **2013** | **2014** | **2015** | **2016** | **2017** | **2018** | **2019** | **2020** | **2021** | **2022** |
| --- | --- | --- | --- | --- | --- | --- | --- | --- | --- | --- | --- | --- | --- | --- | --- |
| Aminoglycosides | % R | French | 0.8 | 2.8 | 2.7 | 0.8 | 0.8 | 2.4 | 2.3 | 1.6 | 3.1 | 2.6 | 1.3 | 6.2 | 2.4 |
| Aminoglycosides | % R | German | 2.4 | 0.8 | 2.1 | 0.7 | 1.4 | 2.3 | 1.6 | 2.1 | 2.7 | 1.7 | 0.5 | 0.5 | 1.7 |
| Aminoglycosides | % R | Italian | 0 | 0 | 7.1 | 9.5 | 0 | 0 | 0 | 0 | 0 | 0 | 0 | 0 | 2.7 |
| Aminoglycosides | Incidence | French | 0.066 | 0.195 | 0.193 | 0.063 | 0.062 | 0.184 | 0.182 | 0.12 | 0.239 | 0.178 | 0.118 | 0.524 | 0.175 |
| Aminoglycosides | Incidence | German | 0.079 | 0.026 | 0.078 | 0.026 | 0.051 | 0.1 | 0.074 | 0.123 | 0.146 | 0.097 | 0.024 | 0.024 | 0.095 |
| Aminoglycosides | Incidence | Italian | 0 | 0 | 0.725 | 0.715 | 0 | 0 | 0 | 0 | 0 | 0 | 0 | 0 | 0.351 |
| Aminoglycosides | N tested | French | 126 | 108 | 110 | 122 | 126 | 124 | 131 | 122 | 130 | 117 | 152 | 146 | 126 |
| Aminoglycosides | N tested | German | 125 | 126 | 146 | 136 | 142 | 177 | 186 | 241 | 224 | 229 | 200 | 212 | 234 |
| Aminoglycosides | N tested | Italian | 23 | 24 | 28 | 21 | 30 | 27 | 22 | 27 | 26 | 32 | 50 | 29 | 37 |
| Carbapenems | % R | French | 11.9 | 7.4 | 13.6 | 11.4 | 16.5 | 16.3 | 13.2 | 11.9 | 8.8 | 18.3 | 10.8 | 14 | 11.4 |
| Carbapenems | % R | German | 7.6 | 6.2 | 5.3 | 8.4 | 11.9 | 10.6 | 9.9 | 8.9 | 9.6 | 11.6 | 9.9 | 9.9 | 7.5 |
| Carbapenems | % R | Italian | 4.3 | 0 | 14.3 | 4.8 | 23.3 | 7.4 | 4.5 | 3.7 | 15.4 | 15.6 | 12 | 13.8 | 13.2 |
| Carbapenems | Incidence | French | 0.989 | 0.52 | 0.964 | 0.884 | 1.305 | 1.226 | 1.091 | 0.901 | 0.716 | 1.303 | 0.999 | 1.223 | 0.874 |
| Carbapenems | Incidence | German | 0.316 | 0.261 | 0.233 | 0.333 | 0.557 | 0.551 | 0.545 | 0.566 | 0.586 | 0.751 | 0.625 | 0.644 | 0.524 |
| Carbapenems | Incidence | Italian | 0.371 | 0 | 1.449 | 0.357 | 2.475 | 0.704 | 0.35 | 0.35 | 1.402 | 1.761 | 2.115 | 1.405 | 1.756 |
| Carbapenems | N tested | French | 126 | 108 | 110 | 123 | 127 | 123 | 136 | 126 | 136 | 120 | 157 | 150 | 132 |
| Carbapenems | N tested | German | 157 | 162 | 171 | 155 | 185 | 208 | 222 | 259 | 251 | 268 | 263 | 273 | 293 |
| Carbapenems | N tested | Italian | 23 | 24 | 28 | 21 | 30 | 27 | 22 | 27 | 26 | 32 | 50 | 29 | 38 |
| Cefepime | % R | French | 1.8 | 4.7 | 7.3 | 6.5 | 7.1 | 11.4 | 5.9 | 4 | 11.8 | 12.5 | 9 | 9.4 | 10 |
| Cefepime | % R | German | 2.2 | 5.4 | 2.5 | 6.8 | 10.1 | 6.9 | 4.3 | 9.1 | 10.7 | 7.7 | 7 | 8.6 | 8 |
| Cefepime | % R | Italian | 100 | 0 | 7.4 | 4.8 | 3.3 | 3.7 | 4.5 | 3.7 | 15.4 | 12.5 | 6 | 6.9 | 10.5 |
| Cefepime | Incidence | French | 0.132 | 0.325 | 0.514 | 0.505 | 0.559 | 0.858 | 0.485 | 0.3 | 0.954 | 0.888 | 0.823 | 0.815 | 0.757 |
| Cefepime | Incidence | German | 0.079 | 0.209 | 0.103 | 0.256 | 0.43 | 0.326 | 0.223 | 0.541 | 0.61 | 0.484 | 0.432 | 0.548 | 0.548 |
| Cefepime | Incidence | Italian | 0.371 | 0 | 0.725 | 0.357 | 0.354 | 0.352 | 0.35 | 0.35 | 1.402 | 1.409 | 1.058 | 0.703 | 1.405 |
| Cefepime | N tested | French | 113 | 107 | 110 | 123 | 127 | 123 | 136 | 125 | 136 | 120 | 156 | 149 | 130 |
| Cefepime | N tested | German | 136 | 148 | 162 | 146 | 169 | 188 | 207 | 243 | 234 | 261 | 257 | 267 | 286 |
| Cefepime | N tested | Italian | 1 | 1 | 27 | 21 | 30 | 27 | 22 | 27 | 26 | 32 | 50 | 29 | 38 |
| Ceftazidime | % R | French | 5 | 8.2 | 11.2 | 10.4 | 10.8 | 17 | 12.8 | 11.9 | 13.3 | 14.7 | 10.8 | 11.9 | 8.3 |
| Ceftazidime | % R | German | 5.7 | 3.7 | 5.3 | 7 | 10.9 | 10 | 7.6 | 10.8 | 12.1 | 8.2 | 7.2 | 7 | 9.3 |
| Ceftazidime | % R | Italian | 8.7 | 4.2 | 7.1 | 4.8 | 6.7 | 11.1 | 9.1 | 7.4 | 7.7 | 9.4 | 6 | 10.3 | 13.2 |
| Ceftazidime | Incidence | French | 0.396 | 0.52 | 0.643 | 0.694 | 0.684 | 1.165 | 0.969 | 0.781 | 0.895 | 0.888 | 0.823 | 0.932 | 0.641 |
| Ceftazidime | Incidence | German | 0.237 | 0.157 | 0.233 | 0.282 | 0.506 | 0.526 | 0.421 | 0.689 | 0.732 | 0.533 | 0.456 | 0.453 | 0.644 |
| Ceftazidime | Incidence | Italian | 0.742 | 0.367 | 0.725 | 0.357 | 0.707 | 1.056 | 0.699 | 0.7 | 0.701 | 1.056 | 1.058 | 1.054 | 1.756 |
| Ceftazidime | N tested | French | 121 | 98 | 89 | 106 | 102 | 112 | 125 | 109 | 113 | 102 | 130 | 135 | 132 |
| Ceftazidime | N tested | German | 157 | 162 | 171 | 157 | 184 | 210 | 224 | 259 | 248 | 268 | 264 | 272 | 289 |
| Ceftazidime | N tested | Italian | 23 | 24 | 28 | 21 | 30 | 27 | 22 | 27 | 26 | 32 | 50 | 29 | 38 |
| Ciprofloxacin | % R | French | 3.2 | 5.6 | 1.8 | 5.7 | 6.3 | 8.1 | 7.4 | 5.6 | 14.8 | 11.7 | 8.3 | 11.3 | 6.8 |
| Ciprofloxacin | % R | German | 2.6 | 6.2 | 5.3 | 9 | 6.5 | 8.6 | 7.1 | 10.4 | 8.8 | 9 | 6.1 | 8.1 | 6.6 |
| Ciprofloxacin | % R | Italian | 8.7 | 16.7 | 14.3 | 19 | 3.3 | 11.1 | 13.6 | 3.7 | 11.5 | 15.6 | 8 | 3.4 | 5.3 |
| Ciprofloxacin | Incidence | French | 0.264 | 0.39 | 0.129 | 0.442 | 0.497 | 0.613 | 0.606 | 0.42 | 1.193 | 0.829 | 0.764 | 0.99 | 0.524 |
| Ciprofloxacin | Incidence | German | 0.105 | 0.261 | 0.233 | 0.358 | 0.304 | 0.451 | 0.397 | 0.664 | 0.537 | 0.581 | 0.384 | 0.524 | 0.453 |
| Ciprofloxacin | Incidence | Italian | 0.742 | 1.469 | 1.449 | 1.429 | 0.354 | 1.056 | 1.049 | 0.35 | 1.051 | 1.761 | 1.41 | 0.351 | 0.703 |
| Ciprofloxacin | N tested | French | 126 | 107 | 110 | 123 | 127 | 123 | 136 | 126 | 135 | 120 | 157 | 150 | 132 |
| Ciprofloxacin | N tested | German | 155 | 162 | 171 | 156 | 185 | 210 | 224 | 260 | 250 | 268 | 264 | 273 | 290 |
| Ciprofloxacin | N tested | Italian | 23 | 24 | 28 | 21 | 30 | 27 | 22 | 27 | 26 | 32 | 50 | 29 | 38 |
| Piperacillin-tazobactam | % R | French | 8.7 | 6.5 | 12.7 | 8.1 | 6.3 | 14.2 | 18.9 | 12.7 | 14 | 16 | 11.8 | 12.2 | 8.3 |
| Piperacillin-tazobactam | % R | German | 4.8 | 10 | 6.2 | 8.8 | 12.9 | 12.6 | 8.9 | 9.9 | 14.6 | 9.1 | 9.5 | 9.8 | 11.6 |
| Piperacillin-tazobactam | % R | Italian | 4.3 | 0 | 7.1 | 4.8 | 10 | 11.1 | 13.6 | 11.1 | 11.5 | 18.8 | 8 | 13.8 | 18.4 |
| Piperacillin-tazobactam | Incidence | French | 0.726 | 0.455 | 0.9 | 0.631 | 0.497 | 1.042 | 1.515 | 0.961 | 1.133 | 1.125 | 1.058 | 1.048 | 0.641 |
| Piperacillin-tazobactam | Incidence | German | 0.185 | 0.392 | 0.259 | 0.333 | 0.557 | 0.652 | 0.471 | 0.615 | 0.854 | 0.533 | 0.553 | 0.596 | 0.763 |
| Piperacillin-tazobactam | Incidence | Italian | 0.371 | 0 | 0.725 | 0.357 | 1.061 | 1.056 | 1.049 | 1.05 | 1.051 | 2.113 | 1.41 | 1.405 | 2.459 |
| Piperacillin-tazobactam | N tested | French | 126 | 108 | 110 | 123 | 127 | 120 | 132 | 126 | 136 | 119 | 153 | 148 | 132 |
| Piperacillin-tazobactam | N tested | German | 146 | 150 | 161 | 148 | 171 | 206 | 214 | 252 | 239 | 243 | 242 | 255 | 276 |
| Piperacillin-tazobactam | N tested | Italian | 23 | 24 | 28 | 21 | 30 | 27 | 22 | 27 | 26 | 32 | 50 | 29 | 38 |
| MDR | % R | French | 2.4 | 4.6 | 5.5 | 4.1 | 4.7 | 7.3 | 5.9 | 4.8 | 6.6 | 10.8 | 5.1 | 6.7 | 4.5 |
| MDR | % R | German | 2.6 | 2.5 | 2.3 | 2.5 | 7.6 | 6.7 | 4.9 | 5 | 8.4 | 4.9 | 4.2 | 2.9 | 2.4 |
| MDR | % R | Italian | 0 | 0 | 3.6 | 9.5 | 3.3 | 7.4 | 4.5 | 0 | 3.8 | 6.2 | 4 | 10.3 | 7.9 |
| MDR | Incidence | French | 0.198 | 0.325 | 0.386 | 0.316 | 0.373 | 0.552 | 0.485 | 0.36 | 0.537 | 0.77 | 0.47 | 0.582 | 0.349 |
| MDR | Incidence | German | 0.105 | 0.104 | 0.103 | 0.102 | 0.354 | 0.351 | 0.273 | 0.32 | 0.513 | 0.315 | 0.264 | 0.191 | 0.167 |
| MDR | Incidence | Italian | 0 | 0 | 0.362 | 0.715 | 0.354 | 0.704 | 0.35 | 0 | 0.35 | 0.704 | 0.705 | 1.054 | 1.054 |
| MDR | N tested | French | 126 | 108 | 110 | 123 | 127 | 123 | 136 | 126 | 136 | 120 | 157 | 150 | 132 |
| MDR | N tested | German | 156 | 162 | 171 | 157 | 185 | 210 | 224 | 260 | 251 | 268 | 264 | 273 | 290 |
| MDR | N tested | Italian | 23 | 24 | 28 | 21 | 30 | 27 | 22 | 27 | 26 | 32 | 50 | 29 | 38 |

French; French-speaking region, German; German-speaking region, Italian; Italian-speaking region, MDR; multidrug-resistant

**Supplementary Table 5** Incidence of *Pseudomonas aeruginosa* bloodstream infections stratified by age group

| **Age group** | **2010** | **2011** | **2012** | **2013** | **2014** | **2015** | **2016** | **2017** | **2018** | **2019** | **2020** | **2021** | **2022** |
| --- | --- | --- | --- | --- | --- | --- | --- | --- | --- | --- | --- | --- | --- |
| **Incidence**  [per 100,000 inhabitants] |  |  |  |  |  |  |  |  |  |  |  |  |  |
| <2 | 7.279 | 3.621 | 3.567 | 8.721 | 10.176 | 8.362 | 1.645 | 11.611 | 9.888 | 6.722 | 0 | 1.632 | 4.897 |
| 2-24 | 0.624 | 1.036 | 0.895 | 0.823 | 0.547 | 0.749 | 0.744 | 1.283 | 0.539 | 0.74 | 1.187 | 0.626 | 0.835 |
| 25-49 | 1.567 | 1.899 | 1.552 | 1.686 | 1.583 | 1.767 | 1.713 | 1.948 | 2.185 | 1.992 | 1.796 | 1.789 | 1.695 |
| 50-64 | 6.476 | 5.904 | 5.426 | 5.919 | 6.37 | 6.397 | 6.748 | 6.468 | 6.204 | 6.432 | 6.513 | 8.383 | 6.922 |
| 65-79 | 19.295 | 16.181 | 17.892 | 14.969 | 16.795 | 17.648 | 19.425 | 19.467 | 19.666 | 19.597 | 24.851 | 22.088 | 22.327 |
| ≥80 | 23.987 | 23.401 | 27.261 | 25.998 | 33.354 | 33.382 | 33.956 | 36.944 | 37.768 | 38.186 | 39.041 | 32.793 | 41.518 |
| **Number of samples** |  |  |  |  |  |  |  |  |  |  |  |  |  |
| <2 | 4 | 2 | 2 | 5 | 6 | 5 | 1 | 7 | 6 | 4 | 0 | 2 | 6 |
| 2-24 | 9 | 15 | 13 | 12 | 8 | 11 | 11 | 19 | 8 | 11 | 17 | 9 | 12 |
| 25-49 | 32 | 39 | 32 | 35 | 33 | 37 | 36 | 41 | 46 | 42 | 38 | 38 | 36 |
| 50-64 | 69 | 64 | 60 | 67 | 74 | 76 | 82 | 80 | 78 | 82 | 84 | 109 | 90 |
| 65-79 | 130 | 112 | 127 | 109 | 125 | 134 | 150 | 153 | 157 | 159 | 205 | 185 | 187 |
| ≥80 | 63 | 63 | 75 | 73 | 96 | 98 | 102 | 113 | 118 | 122 | 126 | 109 | 138 |

**Supplementary Table 6** Resistant *Pseudomonas aeruginosa* bloodstream infections, incidence [N per 100,000 inhabitants] and percentage of resistant isolates [% R] stratified by age group

| **Antibiotic** | **Unit** | **Age group** | **2010** | **2011** | **2012** | **2013** | **2014** | **2015** | **2016** | **2017** | **2018** | **2019** | **2020** | **2021** | **2022** |
| --- | --- | --- | --- | --- | --- | --- | --- | --- | --- | --- | --- | --- | --- | --- | --- |
| Aminoglycosides | % R | <2 | 0 | 0 | 0 | 0 | 0 | 0 | 0 | 0 | 0 | 0 | 0 | 0 | 0 |
| Aminoglycosides | % R | 2-25 | 0 | 7.7 | 7.7 | 0 | 14.3 | 0 | 9.1 | 0 | 25 | 0 | 0 | 16.7 | 8.3 |
| Aminoglycosides | % R | 25-49 | 7.4 | 2.8 | 3.1 | 6.1 | 6.5 | 8.8 | 6.1 | 2.5 | 4.7 | 2.7 | 2.9 | 0 | 0 |
| Aminoglycosides | % R | 50-64 | 1.6 | 1.8 | 5.2 | 3.3 | 0 | 2.8 | 1.3 | 3.9 | 4.2 | 5.1 | 0 | 3 | 1.4 |
| Aminoglycosides | % R | 65-79 | 0.9 | 1.1 | 0.9 | 0 | 0 | 0 | 1.5 | 1.4 | 1.4 | 0.7 | 1.1 | 1.9 | 3.7 |
| Aminoglycosides | % R | ≥80 | 0 | 0 | 3.2 | 0 | 0 | 2.2 | 0 | 1 | 0.9 | 1 | 0 | 3.4 | 0 |
| Aminoglycosides | Incidence | <2 | 0 | 0 | 0 | 0 | 0 | 0 | 0 | 0 | 0 | 0 | 0 | 0 | 0 |
| Aminoglycosides | Incidence | 2-25 | 0 | 0.069 | 0.069 | 0 | 0.068 | 0 | 0.068 | 0 | 0.135 | 0 | 0 | 0.07 | 0.07 |
| Aminoglycosides | Incidence | 25-49 | 0.098 | 0.049 | 0.048 | 0.096 | 0.096 | 0.143 | 0.095 | 0.048 | 0.095 | 0.047 | 0.047 | 0 | 0 |
| Aminoglycosides | Incidence | 50-64 | 0.094 | 0.092 | 0.271 | 0.177 | 0 | 0.168 | 0.082 | 0.243 | 0.239 | 0.314 | 0 | 0.231 | 0.077 |
| Aminoglycosides | Incidence | 65-79 | 0.148 | 0.144 | 0.141 | 0 | 0 | 0 | 0.259 | 0.254 | 0.251 | 0.123 | 0.242 | 0.358 | 0.716 |
| Aminoglycosides | Incidence | ≥80 | 0 | 0 | 0.727 | 0 | 0 | 0.681 | 0 | 0.327 | 0.32 | 0.313 | 0 | 0.903 | 0 |
| Carbapenems | % R | <2 | 25 | 0 | 0 | 0 | 16.7 | 0 | 0 | 0 | 0 | 0 | 0 | 0 | 0 |
| Carbapenems | % R | 2-25 | 22.2 | 13.3 | 15.4 | 41.7 | 12.5 | 36.4 | 18.2 | 15.8 | 37.5 | 36.4 | 17.6 | 22.2 | 25 |
| Carbapenems | % R | 25-49 | 12.5 | 18.4 | 18.8 | 20 | 18.2 | 35.1 | 22.2 | 9.8 | 13 | 23.8 | 13.2 | 21.1 | 16.7 |
| Carbapenems | % R | 50-64 | 13.2 | 6.2 | 13.3 | 14.9 | 23 | 16 | 13.6 | 15 | 9 | 16 | 15.5 | 13.8 | 13.3 |
| Carbapenems | % R | 65-79 | 7.7 | 3.6 | 7.9 | 2.8 | 11.2 | 9.1 | 10.7 | 9.9 | 11.5 | 10.6 | 8.3 | 10.8 | 8.1 |
| Carbapenems | % R | ≥80 | 3.2 | 1.6 | 2.7 | 4.2 | 11.5 | 3.1 | 4 | 4.4 | 5.1 | 11.5 | 8.8 | 6.4 | 4.5 |
| Carbapenems | Incidence | <2 | 1.82 | 0 | 0 | 0 | 1.696 | 0 | 0 | 0 | 0 | 0 | 0 | 0 | 0 |
| Carbapenems | Incidence | 2-25 | 0.139 | 0.138 | 0.138 | 0.343 | 0.068 | 0.272 | 0.135 | 0.203 | 0.202 | 0.269 | 0.209 | 0.139 | 0.209 |
| Carbapenems | Incidence | 25-49 | 0.196 | 0.341 | 0.291 | 0.337 | 0.288 | 0.621 | 0.381 | 0.19 | 0.285 | 0.474 | 0.236 | 0.377 | 0.282 |
| Carbapenems | Incidence | 50-64 | 0.845 | 0.369 | 0.723 | 0.883 | 1.463 | 1.01 | 0.905 | 0.97 | 0.557 | 1.02 | 1.008 | 1.154 | 0.923 |
| Carbapenems | Incidence | 65-79 | 1.484 | 0.578 | 1.409 | 0.412 | 1.881 | 1.58 | 2.072 | 1.909 | 2.255 | 2.095 | 2.061 | 2.388 | 1.791 |
| Carbapenems | Incidence | ≥80 | 0.762 | 0.371 | 0.727 | 1.068 | 3.822 | 1.022 | 1.332 | 1.635 | 1.92 | 4.382 | 3.408 | 2.106 | 1.805 |
| Cefepime | % R | <2 | 0 | 0 | 0 | 0 | 0 | 33.3 | 0 | 0 | 0 | 0 | 0 | 0 | 0 |
| Cefepime | % R | 2-25 | 0 | 16.7 | 0 | 18.2 | 16.7 | 22.2 | 11.1 | 12.5 | 37.5 | 10 | 17.6 | 11.1 | 16.7 |
| Cefepime | % R | 25-49 | 0 | 8.6 | 9.4 | 20 | 18.2 | 13.9 | 11.4 | 9.8 | 19.6 | 16.7 | 10.8 | 18.4 | 11.4 |
| Cefepime | % R | 50-64 | 5.1 | 7 | 11.7 | 7.9 | 9.9 | 9.6 | 8.1 | 11.7 | 5.6 | 16 | 10.8 | 11.9 | 11.5 |
| Cefepime | % R | 65-79 | 2.8 | 3.2 | 0.8 | 2.8 | 7.6 | 5.6 | 4.1 | 8.4 | 9.2 | 4.5 | 5.9 | 5 | 9.8 |
| Cefepime | % R | ≥80 | 0 | 1.8 | 4.1 | 2.9 | 4.3 | 6.5 | 1 | 0.9 | 13 | 9.1 | 5.7 | 8.4 | 4.6 |
| Cefepime | Incidence | <2 | 0 | 0 | 0 | 0 | 0 | 1.672 | 0 | 0 | 0 | 0 | 0 | 0 | 0 |
| Cefepime | Incidence | 2-25 | 0 | 0.138 | 0 | 0.137 | 0.068 | 0.136 | 0.068 | 0.135 | 0.202 | 0.067 | 0.209 | 0.07 | 0.139 |
| Cefepime | Incidence | 25-49 | 0 | 0.146 | 0.145 | 0.337 | 0.288 | 0.239 | 0.19 | 0.19 | 0.428 | 0.332 | 0.189 | 0.33 | 0.188 |
| Cefepime | Incidence | 50-64 | 0.282 | 0.369 | 0.633 | 0.442 | 0.603 | 0.589 | 0.494 | 0.728 | 0.318 | 1.02 | 0.698 | 1 | 0.769 |
| Cefepime | Incidence | 65-79 | 0.445 | 0.433 | 0.141 | 0.412 | 1.209 | 0.922 | 0.777 | 1.527 | 1.754 | 0.863 | 1.455 | 1.075 | 2.149 |
| Cefepime | Incidence | ≥80 | 0 | 0.371 | 1.09 | 0.712 | 1.39 | 2.044 | 0.333 | 0.327 | 4.801 | 3.443 | 2.169 | 2.708 | 1.805 |
| Ceftazidime | % R | <2 | 0 | 0 | 0 | 0 | 0 | 20 | 0 | 0 | 0 | 0 | 0 | 0 | 0 |
| Ceftazidime | % R | 2-25 | 11.1 | 13.3 | 15.4 | 16.7 | 12.5 | 27.3 | 9.1 | 10.5 | 25 | 18.2 | 0 | 11.1 | 16.7 |
| Ceftazidime | % R | 25-49 | 6.2 | 11.1 | 10 | 20.6 | 22.6 | 36.1 | 16.7 | 12.8 | 20.9 | 16.7 | 13.2 | 14.3 | 11.4 |
| Ceftazidime | % R | 50-64 | 8.8 | 9.8 | 12.7 | 7.6 | 11.9 | 11.4 | 15 | 15.6 | 9.6 | 15.4 | 13.8 | 13.1 | 13.6 |
| Ceftazidime | % R | 65-79 | 4.7 | 1.8 | 5 | 4.9 | 8.7 | 9.2 | 6.2 | 10.4 | 12.1 | 6 | 8.9 | 5.6 | 9.7 |
| Ceftazidime | % R | ≥80 | 3.3 | 1.6 | 4.3 | 6.2 | 7.9 | 6.2 | 7.1 | 8.3 | 10.2 | 8.6 | 2.5 | 7.6 | 5.3 |
| Ceftazidime | Incidence | <2 | 0 | 0 | 0 | 0 | 0 | 1.672 | 0 | 0 | 0 | 0 | 0 | 0 | 0 |
| Ceftazidime | Incidence | 2-25 | 0.069 | 0.138 | 0.138 | 0.137 | 0.068 | 0.204 | 0.068 | 0.135 | 0.135 | 0.134 | 0 | 0.07 | 0.139 |
| Ceftazidime | Incidence | 25-49 | 0.098 | 0.195 | 0.145 | 0.337 | 0.336 | 0.621 | 0.286 | 0.238 | 0.428 | 0.332 | 0.236 | 0.235 | 0.188 |
| Ceftazidime | Incidence | 50-64 | 0.563 | 0.553 | 0.633 | 0.442 | 0.689 | 0.673 | 0.988 | 0.97 | 0.557 | 0.941 | 0.853 | 1.077 | 0.923 |
| Ceftazidime | Incidence | 65-79 | 0.891 | 0.289 | 0.845 | 0.687 | 1.344 | 1.58 | 1.166 | 1.909 | 2.255 | 1.109 | 2.061 | 1.194 | 2.149 |
| Ceftazidime | Incidence | ≥80 | 0.762 | 0.371 | 1.09 | 1.425 | 2.432 | 2.044 | 2.33 | 2.942 | 3.521 | 3.13 | 0.93 | 2.407 | 2.106 |
| Ciprofloxacin | % R | <2 | 0 | 0 | 0 | 0 | 0 | 0 | 0 | 0 | 0 | 0 | 0 | 50 | 0 |
| Ciprofloxacin | % R | 2-25 | 11.1 | 6.7 | 7.7 | 16.7 | 12.5 | 27.3 | 18.2 | 10.5 | 25 | 27.3 | 17.6 | 11.1 | 16.7 |
| Ciprofloxacin | % R | 25-49 | 0 | 7.9 | 12.5 | 14.3 | 15.2 | 24.3 | 13.9 | 9.8 | 17.8 | 14.3 | 13.2 | 23.7 | 5.7 |
| Ciprofloxacin | % R | 50-64 | 5.9 | 7.9 | 5 | 10.6 | 10.8 | 4 | 7.3 | 8.8 | 10.4 | 6.1 | 9.5 | 8.3 | 2.3 |
| Ciprofloxacin | % R | 65-79 | 3.8 | 5.4 | 3.9 | 4.6 | 4 | 5.2 | 5.3 | 9.8 | 8.3 | 9.4 | 3.9 | 8.1 | 10.2 |
| Ciprofloxacin | % R | ≥80 | 0 | 7.9 | 2.7 | 8.2 | 2.1 | 9.2 | 7.8 | 6.2 | 11.9 | 11.5 | 7.1 | 4.6 | 3.8 |
| Ciprofloxacin | Incidence | <2 | 0 | 0 | 0 | 0 | 0 | 0 | 0 | 0 | 0 | 0 | 0 | 0.816 | 0 |
| Ciprofloxacin | Incidence | 2-25 | 0.069 | 0.069 | 0.069 | 0.137 | 0.068 | 0.204 | 0.135 | 0.135 | 0.135 | 0.202 | 0.209 | 0.07 | 0.139 |
| Ciprofloxacin | Incidence | 25-49 | 0 | 0.146 | 0.194 | 0.241 | 0.24 | 0.43 | 0.238 | 0.19 | 0.38 | 0.285 | 0.236 | 0.424 | 0.094 |
| Ciprofloxacin | Incidence | 50-64 | 0.375 | 0.461 | 0.271 | 0.618 | 0.689 | 0.253 | 0.494 | 0.566 | 0.636 | 0.392 | 0.62 | 0.692 | 0.154 |
| Ciprofloxacin | Incidence | 65-79 | 0.742 | 0.867 | 0.704 | 0.687 | 0.672 | 0.922 | 1.036 | 1.909 | 1.628 | 1.849 | 0.97 | 1.791 | 2.269 |
| Ciprofloxacin | Incidence | ≥80 | 0 | 1.857 | 0.727 | 2.137 | 0.695 | 3.066 | 2.663 | 2.289 | 4.481 | 4.382 | 2.789 | 1.504 | 1.504 |
| Piperacillin-tazobactam | % R | <2 | 0 | 0 | 0 | 0 | 16.7 | 20 | 0 | 0 | 0 | 0 | 0 | 0 | 0 |
| Piperacillin-tazobactam | % R | 2-25 | 16.7 | 26.7 | 15.4 | 16.7 | 25 | 36.4 | 9.1 | 15.8 | 37.5 | 27.3 | 23.5 | 12.5 | 16.7 |
| Piperacillin-tazobactam | % R | 25-49 | 6.7 | 10.8 | 12.5 | 20.6 | 15.2 | 27 | 22.2 | 10 | 22.7 | 20.5 | 13.5 | 19.4 | 18.8 |
| Piperacillin-tazobactam | % R | 50-64 | 11.9 | 8.1 | 11.9 | 9.2 | 13 | 13.5 | 16.5 | 16.5 | 9 | 13.8 | 17.3 | 15.1 | 16.7 |
| Piperacillin-tazobactam | % R | 65-79 | 5.6 | 4.7 | 8.1 | 4.7 | 8.4 | 8.4 | 9.1 | 9.3 | 14.4 | 6.1 | 7.3 | 7.9 | 11.2 |
| Piperacillin-tazobactam | % R | ≥80 | 1.6 | 6.7 | 4.3 | 5.7 | 6.5 | 10.5 | 12.2 | 9.1 | 13.4 | 14.2 | 6.7 | 8.8 | 6 |
| Piperacillin-tazobactam | Incidence | <2 | 0 | 0 | 0 | 0 | 1.696 | 1.672 | 0 | 0 | 0 | 0 | 0 | 0 | 0 |
| Piperacillin-tazobactam | Incidence | 2-25 | 0.069 | 0.276 | 0.138 | 0.137 | 0.137 | 0.272 | 0.068 | 0.203 | 0.202 | 0.202 | 0.279 | 0.07 | 0.139 |
| Piperacillin-tazobactam | Incidence | 25-49 | 0.098 | 0.195 | 0.194 | 0.337 | 0.24 | 0.478 | 0.381 | 0.19 | 0.475 | 0.38 | 0.236 | 0.33 | 0.282 |
| Piperacillin-tazobactam | Incidence | 50-64 | 0.751 | 0.461 | 0.633 | 0.53 | 0.775 | 0.842 | 1.07 | 1.051 | 0.557 | 0.863 | 1.085 | 1.231 | 1.077 |
| Piperacillin-tazobactam | Incidence | 65-79 | 1.039 | 0.722 | 1.409 | 0.687 | 1.344 | 1.449 | 1.684 | 1.781 | 2.756 | 1.109 | 1.697 | 1.672 | 2.388 |
| Piperacillin-tazobactam | Incidence | ≥80 | 0.381 | 1.486 | 1.09 | 1.425 | 2.085 | 3.406 | 3.995 | 3.269 | 4.801 | 5.008 | 2.479 | 2.708 | 2.407 |
| MDR | % R | <2 | 0 | 0 | 0 | 0 | 0 | 0 | 0 | 0 | 0 | 0 | 0 | 0 | 0 |
| MDR | % R | 2-25 | 0 | 6.7 | 7.7 | 8.3 | 12.5 | 27.3 | 9.1 | 10.5 | 37.5 | 27.3 | 5.9 | 11.1 | 16.7 |
| MDR | % R | 25-49 | 3.2 | 10.5 | 6.2 | 11.4 | 12.1 | 21.6 | 13.9 | 7.3 | 8.7 | 16.7 | 10.5 | 10.5 | 8.6 |
| MDR | % R | 50-64 | 4.4 | 6.2 | 10 | 4.5 | 12.2 | 8 | 6.1 | 6.2 | 9 | 9.8 | 9.5 | 7.3 | 3.4 |
| MDR | % R | 65-79 | 2.3 | 0 | 0.8 | 0.9 | 4 | 3.7 | 3.3 | 4.6 | 7.6 | 1.3 | 2.9 | 1.6 | 3.8 |
| MDR | % R | ≥80 | 0 | 0 | 1.3 | 2.7 | 2.1 | 3.1 | 3.9 | 1.8 | 4.2 | 6.6 | 1.6 | 4.6 | 0.8 |
| MDR | Incidence | <2 | 0 | 0 | 0 | 0 | 0 | 0 | 0 | 0 | 0 | 0 | 0 | 0 | 0 |
| MDR | Incidence | 2-25 | 0 | 0.069 | 0.069 | 0.069 | 0.068 | 0.204 | 0.068 | 0.135 | 0.202 | 0.202 | 0.07 | 0.07 | 0.139 |
| MDR | Incidence | 25-49 | 0.049 | 0.195 | 0.097 | 0.193 | 0.192 | 0.382 | 0.238 | 0.143 | 0.19 | 0.332 | 0.189 | 0.188 | 0.141 |
| MDR | Incidence | 50-64 | 0.282 | 0.369 | 0.543 | 0.265 | 0.775 | 0.505 | 0.411 | 0.404 | 0.557 | 0.628 | 0.62 | 0.615 | 0.231 |
| MDR | Incidence | 65-79 | 0.445 | 0 | 0.141 | 0.137 | 0.672 | 0.659 | 0.648 | 0.891 | 1.503 | 0.246 | 0.727 | 0.358 | 0.836 |
| MDR | Incidence | ≥80 | 0 | 0 | 0.363 | 0.712 | 0.695 | 1.022 | 1.332 | 0.654 | 1.6 | 2.504 | 0.62 | 1.504 | 0.301 |

MDR; multidrug-resistant

**Supplementary Table 7** Incidence of *Pseudomonas aeruginosa* bloodstream infections [N per 100,000 inhabitants] stratified by sex

| **Sex** | **2010** | **2011** | **2012** | **2013** | **2014** | **2015** | **2016** | **2017** | **2018** | **2019** | **2020** | **2021** | **2022** |
| --- | --- | --- | --- | --- | --- | --- | --- | --- | --- | --- | --- | --- | --- |
| **Incidence**  [N per 100,000 inhabitants] |  |  |  |  |  |  |  |  |  |  |  |  |  |
| Female | 3.201 | 2.817 | 3.628 | 3.104 | 3.376 | 4.052 | 3.378 | 5.013 | 3.824 | 4.223 | 4.812 | 4.195 | 4.582 |
| Male | 7.947 | 7.784 | 7.335 | 7.449 | 8.47 | 8.303 | 9.561 | 8.845 | 9.953 | 9.68 | 10.627 | 10.54 | 10.704 |
| **Number of samples** |  |  |  |  |  |  |  |  |  |  |  |  |  |
| Female | 90 | 80 | 104 | 90 | 99 | 120 | 101 | 151 | 116 | 129 | 148 | 130 | 142 |
| Male | 217 | 215 | 205 | 211 | 243 | 241 | 281 | 262 | 297 | 291 | 322 | 322 | 327 |

**Supplementary Table 8** Resistant *Pseudomonas aeruginosa* bloodstream infections, incidence [N per 100,000 inhabitants] and percentage of resistant isolates [% R] stratified by sex

| **Antibiotic** | **Unit** | **Sex** | **2010** | **2011** | **2012** | **2013** | **2014** | **2015** | **2016** | **2017** | **2018** | **2019** | **2020** | **2021** | **2022** |
| --- | --- | --- | --- | --- | --- | --- | --- | --- | --- | --- | --- | --- | --- | --- | --- |
| Aminoglycosides | % R | f | 2.4 | 2.7 | 3.2 | 1.2 | 0 | 2.8 | 1.1 | 0 | 1.9 | 0 | 0.8 | 3.7 | 0.9 |
| Aminoglycosides | % R | m | 1.1 | 1.1 | 2.6 | 1.5 | 1.4 | 1.8 | 2 | 2.8 | 2.9 | 2.7 | 0.7 | 2.2 | 2.5 |
| Aminoglycosides | Incidence | f | 0.071 | 0.07 | 0.105 | 0.034 | 0 | 0.101 | 0.033 | 0 | 0.066 | 0 | 0.033 | 0.129 | 0.032 |
| Aminoglycosides | Incidence | m | 0.073 | 0.072 | 0.179 | 0.106 | 0.105 | 0.138 | 0.17 | 0.236 | 0.268 | 0.233 | 0.066 | 0.196 | 0.229 |
| Carbapenems | % R | f | 10.1 | 5 | 10.6 | 10 | 12.1 | 15 | 7 | 8.6 | 10.3 | 12.4 | 8.7 | 8.5 | 5 |
| Carbapenems | % R | m | 8.8 | 6.5 | 8.3 | 9.1 | 15.6 | 10.9 | 12.1 | 10 | 9.4 | 14.4 | 11.2 | 12.7 | 10.8 |
| Carbapenems | Incidence | f | 0.32 | 0.141 | 0.384 | 0.31 | 0.409 | 0.608 | 0.234 | 0.432 | 0.396 | 0.524 | 0.423 | 0.355 | 0.226 |
| Carbapenems | Incidence | m | 0.696 | 0.507 | 0.608 | 0.671 | 1.325 | 0.896 | 1.157 | 0.878 | 0.938 | 1.397 | 1.188 | 1.342 | 1.146 |
| Cefepime | % R | f | 2.7 | 7 | 3 | 4.5 | 7.5 | 9.5 | 3.2 | 4.2 | 11.8 | 11 | 6.1 | 7.8 | 8.8 |
| Cefepime | % R | m | 2.3 | 4.3 | 5.6 | 7.4 | 8.6 | 7.7 | 5.6 | 8.7 | 11.2 | 8.7 | 8.3 | 9.1 | 8.8 |
| Cefepime | Incidence | f | 0.071 | 0.176 | 0.105 | 0.138 | 0.239 | 0.371 | 0.1 | 0.199 | 0.429 | 0.458 | 0.293 | 0.323 | 0.387 |
| Cefepime | Incidence | m | 0.146 | 0.29 | 0.394 | 0.53 | 0.697 | 0.586 | 0.51 | 0.743 | 1.072 | 0.832 | 0.858 | 0.949 | 0.917 |
| Ceftazidime | % R | f | 8 | 5.3 | 6.2 | 4.9 | 11.1 | 13.7 | 9 | 8.3 | 10.1 | 10.4 | 9.5 | 7.2 | 8.6 |
| Ceftazidime | % R | m | 4.7 | 5.3 | 7.8 | 9.4 | 10.2 | 11.6 | 9.6 | 12.4 | 12.9 | 9.7 | 7.5 | 9.3 | 9.7 |
| Ceftazidime | Incidence | f | 0.249 | 0.141 | 0.209 | 0.138 | 0.341 | 0.54 | 0.301 | 0.398 | 0.363 | 0.426 | 0.423 | 0.29 | 0.387 |
| Ceftazidime | Incidence | m | 0.366 | 0.398 | 0.537 | 0.671 | 0.802 | 0.93 | 0.885 | 1.046 | 1.206 | 0.898 | 0.759 | 0.949 | 1.015 |
| Ciprofloxacin | % R | f | 2.2 | 1.3 | 2.9 | 10.1 | 2 | 11.7 | 5 | 4.6 | 6.1 | 10.8 | 9.4 | 7.7 | 3.6 |
| Ciprofloxacin | % R | m | 3.7 | 8.9 | 5.9 | 7.6 | 7.8 | 7.1 | 8.5 | 10.7 | 12.8 | 10 | 5.9 | 9.3 | 7.8 |
| Ciprofloxacin | Incidence | f | 0.071 | 0.035 | 0.105 | 0.31 | 0.068 | 0.473 | 0.167 | 0.232 | 0.231 | 0.458 | 0.455 | 0.323 | 0.161 |
| Ciprofloxacin | Incidence | m | 0.293 | 0.688 | 0.429 | 0.565 | 0.662 | 0.586 | 0.817 | 0.945 | 1.274 | 0.965 | 0.627 | 0.982 | 0.818 |
| Piperacillin-tazobactam | % R | f | 6.9 | 9 | 6.9 | 4.6 | 11.6 | 9.4 | 12.4 | 7.4 | 12.7 | 11.3 | 9.9 | 8.2 | 10.3 |
| Piperacillin-tazobactam | % R | m | 6.2 | 7.4 | 9.6 | 9.8 | 9.4 | 14.8 | 12.9 | 12.8 | 14.8 | 12.2 | 10.2 | 11.9 | 11.6 |
| Piperacillin-tazobactam | Incidence | f | 0.213 | 0.247 | 0.244 | 0.138 | 0.375 | 0.371 | 0.401 | 0.365 | 0.462 | 0.458 | 0.455 | 0.323 | 0.452 |
| Piperacillin-tazobactam | Incidence | m | 0.476 | 0.543 | 0.68 | 0.706 | 0.767 | 1.206 | 1.191 | 1.114 | 1.441 | 1.098 | 1.023 | 1.211 | 1.178 |
| MDR | % R | f | 3.4 | 2.5 | 3.8 | 2.2 | 4 | 6.7 | 4 | 2 | 6.9 | 6.9 | 4 | 4.6 | 2.2 |
| MDR | % R | m | 1.9 | 3.3 | 3.4 | 4.3 | 7 | 7.1 | 5.7 | 6.1 | 7.7 | 6.6 | 4.7 | 4.7 | 4 |
| MDR | Incidence | f | 0.107 | 0.07 | 0.14 | 0.069 | 0.136 | 0.27 | 0.134 | 0.1 | 0.264 | 0.295 | 0.195 | 0.194 | 0.097 |
| MDR | Incidence | m | 0.146 | 0.253 | 0.25 | 0.318 | 0.593 | 0.586 | 0.544 | 0.54 | 0.771 | 0.632 | 0.495 | 0.491 | 0.426 |

f; female, m; male, MDR; multidrug-resistant

**Supplementary Table 9** Results of the Poisson regression models describing the overall incidence of *Pseudomonas aeruginosa* bloodstream infections and the incidence of resistant isolates considering the linguistic regions

| **Predictor variable** | **Overall Incidence** | **Aminoglycosides** | **Carbapenems** | **Cefepime** | **Ceftazidime** | **Ciprofloxacin** | **Piperacillin-tazobactam** | | **MDR** | |
| --- | --- | --- | --- | --- | --- | --- | --- | --- | --- | --- |
| Yearly increase | - | - | - | *p* = 0.001 | - | *p* < 0.001 | - | - | |  |
| **Incidence compared to the French-speaking region** | | |  |  |  |  |  |  | |  |
| German < French | *p* < 0.001 | - | *p* = 0.036 | - | - | - | - | - | |  |
| German > French | - | - | - | - | - | - | - | - | |  |
| Italian < French | - | - | - | - | - | - | *p* = 0.025 |  | |  |
| Italian > French | - | - | - | - | - | *p* = 0.026 | - |  | |  |
| **Yearly increase compared to the increase in the French-speaking region** | | | |  |  |  |  |  | |  |
| ↑ German > ↑ French | *p* < 0.001 | - | *p* = 0.037 | - | - | - | - | - | |  |
| ↑ Italian > ↑ French | - | - | - | - | - | - | *p* = 0.024 | - | |  |

French; French-speaking region, German; German-speaking region, Italian; Italian-speaking region, ↑; increase, -; not significant

**Supplementary Table 10** Results of the multiple logistic regression models describing the percentage of resistant isolates among *Pseudomonas aeruginosa* bloodstream infections considering the linguistic regions

| **Predictor variable** | **Aminoglycosides** | **Carbapenems** | **Cefepime** | **Ceftazidime** | **Ciprofloxacin** | **Piperacillin-tazobactam** | **MDR** |
| --- | --- | --- | --- | --- | --- | --- | --- |
| Yearly increase | - | - | *p* = 0.014 | - | *p* = 0.027 | - | - |
| **Resistance rate compared to the French-speaking region** | | |  |  |  |  |  |
| Italian > French | - | - | - | - | *p* = 0.002 | - | - |
| German > French | - | - | - | - | - | - | - |
| **Yearly increase compared to the increase in the French-speaking region** | | | |  |  |  |  |
| ↑ Italian < ↑ French | - | - | - | - | *p* = 0.002 | - |  |
| ↑ Italian > ↑ French | - | - | - | - | - | *p* = 0.028 | - |
| ↑ German < ↑ French | - | - | - | - | - | - | - |
| ↑ German > ↑ French | - | - | - | - | - | - | - |

French; French-speaking region, German; German-speaking region, Italian; Italian-speaking region, ↑; increase, -; not significant
